# Analysis of m6A Methylation Modification Patterns and Tumor Immune Microenvironment in Breast Cancer

**DOI:** 10.3389/fcell.2022.785058

**Published:** 2022-02-01

**Authors:** Menglu Dong, Wenzhuang Shen, Guang Yang, Zhifang Yang, Xingrui Li

**Affiliations:** ^1^ Department of Thyroid and Breast Surgery, Tongji Hospital, Tongji Medical College of Huazhong University of Science and Technology, Wuhan, China; ^2^ Department of Thoracic Surgery, Tongji Hospital, Tongji Medical College, Huazhong University of Science and Technology, Wuhan, China

**Keywords:** M6A, tumor microenvironment, breast cancer, immunotherapy, mutation burden

## Abstract

Increasing evidence indicates that the abnormal expression of N6-methyladenosine (m6A) modification is closely related to the epigenetic regulation of immune response in breast cancer (BC). However, the potential roles of m6A modification in the tumor microenvironment (TME) of BC remain unclear. For addressing this issue, we comprehensively analyzed the m6A modification patterns in 983 samples and correlated these modification patterns with TME immune cell infiltration, based on 23 kinds of m6A regulators. Principal component analysis (PCA) was used to construct the m6A scoring system to quantify the modification pattern of m6A of BC individuals. Consequently, three different m6A modification patterns were identified, and the infiltrating characteristics of TME cells were consistent with the three immune phenotypes, including immune rejection, immune inflammation, and immune desert. Besides, our analysis showed that the prognosis of patients could be predicted by evaluating the m6A modification pattern in the tumor. The low m6Ascore corresponded to increased mutation burden and immune activation, while stroma activation and lack of immune infiltration were observed in high m6Ascore subtypes. In addition, a low m6Ascore was associated with enhanced response to anti-PD-1/PD-L1 immunotherapy. In conclusion, the m6A modification pattern was closely related to the BC immune landscape. This well-validated score model of the m6A modification patterns will provide a valuable tool to depict the tumor immune state and guide effective tumor immunotherapy for combating BC.

## Introduction

As the third layer of epigenetics, N6-methyladenosine (m6A) is considered to be the most common modification type in mRNAs and long non-coding RNAs (lncRNAs) (Alarcón et al., 2015). M6A methylation can affect various aspects of RNA metabolism, including RNA translocation, splicing, stability, and translation into proteins. Similar to DNA and protein modification, m6A modification is a dynamic and reversible process mediated by three different types of regulatory proteins: methyltransferases (writers), demethylases (erasers), and m6A-binding proteins (readers). The m6A methyltransferases mainly include RBM15, ZC3H13, METTL3, METTL14, WTAP, and KIAA1429, which are involved in forming m6A methylation (Zhao et al., 2017). The demethylases, represented by FTO and ALKBH5, can mediate the m6A removal process that selectively removes methyl codes from specific mRNAs. In addition, the specific m6A-binding proteins, consisting of the YTHDF family (YTHDF1/2/3), YTHDC1/2, eukaryotic initiation factors (eIF or EIF1A), and nuclear heterogeneous riboprotein family (HNRNPA2B1 and HNRNPC), are m6A readers that can recognize and bind to the m6A methylation sites of in RNA, thereby affecting the m6A functions. Besides, m6A is an important RNA modification that regulates various key cellular processes (Meyer and Jaffrey, 2017). The aberrant levels of m6A modification and its regulators are closely related to the dysregulation of various biological processes, including cell proliferation/differentiation, stem cell renewal, malignant progression, impaired self-renewal ability, and abnormal immune regulation (Natalia et al., 2018). The present studies on the m6A regulators will help interpret the impact and mechanism of m6A modification in post-transcriptional regulation.

Nowadays, it is well-documented that the abnormal expressions of m6A methylation modification potentially contribute to the activation and inhibition of multiple signal molecules in cancers, including bladder cancer, gastric cancer, hepatocellular carcinoma (HCC), and breast cancer (BC). The tumor microenvironment (TME) in BC is a dynamic entity composed of widely diverse cancerous and non-cancerous cells, including cancer-related fibroblasts (CAF), adipocytes, and infiltrating immune cells (myeloid cells and lymphocytes) (Fang and Declerck, 2013). It is noteworthy that tumor cells, through direct or indirect interactions with other TME components, participate in various biological behavior changes, such as inhibition of apoptosis, avoidance of hypoxia, induction of proliferation and angiogenesis, and induction of immune tolerance. Immunotherapy, represented by targeting immune checkpoint blockade (ICB), programmed cell death protein 1 (PD-1)/programmed cell death-ligand 1 (PD-L1), and cytotoxic T-lymphocyte-associated antigen 4 (CTLA-4) immunological checkpoint blockade, brings significant clinical efficacy in a small number of patients with a sustained response. However, most clinical patients have little or no benefit (Fang and Declerck, 2013), mainly because the composition and density of immune cells in the TME profoundly affect the tumor progression and the efficiency of anti-cancer therapies. TME plays an increasingly important role in tumor progression, immune escape, and immunotherapy response. The characteristics of immune cell infiltration in the complicated TME and their clinic pathological significance have been successfully utilized for predicting patient response to immunotherapy (Quail and Joyce, 2013; Ali et al., 2016). Comprehensive analysis of the heterogeneity and complexity of TME can help identify different tumor immune phenotypes, predict the response to immunotherapy, and develop new therapeutic targets (Wang et al., 2019).

M6A modification has provided possibilities for changing the fate of cancer cells and immune cells, consequently controlling cancer progression. Numerous studies have revealed a specific correlation between m6A regulators and TME infiltrating immune cells. For example, Han et al. reported that YTHDF1 could improve the translation efficiency of lysosomal cathepsin in dendritic cells (DCs) by binding to the m6A-methylated lysosomal protease transcript (Han et al., 2019). In addition, the inhibition of cathepsins in DC improves the ability of tumor antigen cross-presentation, thus enhancing the antitumor response of tumor-infiltrating CD8^+^ T cells. It was intriguing that the inhibition of YTHDF1 significantly improved the therapeutic effect of anti-PD-L1 blockers (Li et al., 2019). Wang et al. also confirmed that METTL3 could improve the translation level of some immune transcripts by mediating m6A modification and physiologically promoted DC activation and T-cell response (Wang et al., 2019). In addition, the loss of METTTL3 in T cells disrupted T-cell homeostasis and differentiation (Li et al., 2017). Hence, the tumor progression is characterized by the interaction of multiple m6A regulators, potentially associated with clinical features, prognosis, immune cell infiltration, and even immunotherapy efficacy in BC. However, most previous studies have focused on one or a limited number of m6A regulators and cell types rather than the global m6A regulator levels.

Therefore, there is an urgent need to comprehensively and cautiously investigate the prognostic value of the m6A regulatory factor and its relationship with infiltrating characteristics of TME in BC. Herein, using The Cancer Genome Atlas (TCGA) and Gene-Expression Omnibus (GEO), we mainly analyzed the correlation between the modification pattern of m6A based on 23 m6A regulators and TME cell infiltration characteristics. We here identified three different patterns of m6A modification and found that they had different prognosis and infiltrating characteristics of TME cells, indicating that m6A modification is a crucial player in TME of BC. Therefore, we successfully constructed a scoring system to quantify the modification pattern of m6A in individual BC patients based on the expression of m6A regulatory factors.

## Methods

### Breast Cancer Dataset and Processing

Common gene expression data and complete clinical records of BC patients were retrieved from TCGA and the GEO database (http://www.ncbi.nlm.nih.gov/geo/) (Xin et al., 2020). The BC patients without survival information were excluded. In this study, a total of six eligible BC GEO cohorts [GSE21653 (Sabatier et al., 2011), GSE24450 (Heikkinen et al., 2011), GSE42568 (Clarke et al., 2013), GSE45255 (Nagalla et al., 2013), GSE51783 (Zhao et al., 2014), and GSE61304 (Grinchuk et al., 2015)] with complete prognostic information and mRNA sequence information and The Cancer Genome Atlas-BC (TCGA-BC) were included together for further analysis (Supplementary Table S1). The averaging method of Affy and simpleafty was used to adjust background and quantile normalization. Our research flowchart is shown in Supplementary Figure S1. We downloaded the RNA sequencing data (FPKM value) from the Genomic Data Commons (GDC, https://portal.gdc.cancer.gov/) and used the R package TCGAbiolinks for comprehensive analysis. TCGA bio links package was a software package specially developed for GDC data analysis (Colaprico et al., 2016). The somatic mutation dataset was also from the TCGA database, and the R (version 3.6.3) and R biological conductor packages were used to analyze the data.

### Consensus Clustering Analysis for 23 m6A Regulators

A total of 23 m6A RNA methylation regulators were selected from the published literature (Yang et al., 2018; Shi et al., 2019). Next, based on the expression levels of 23 m6A regulators, we used unsupervised cluster analysis to classify the patients into three m6A modification modes with optimal k value, used the ConsensClusterPlus R software package to cluster the patients, and performed cycle computation 1,000 for guaranteeing the stability of classification (Wilkerson and Hayes, 2010). The overall survival (OS) between different clusters was calculated by the Kaplan–Meier statistical method.

### Gene-Set Variation Analysis and Functional Annotation

GSVA is a non-parametric and unsupervised method commonly used to estimate the path variation and the activity change of biological processes in samples of expression datasets (Hänzelmann et al., 2013). Then, the “GSVA” R package was used to analyze the GSVA enrichment of genes and studied the enrichment of biological processes between different m6A modification modes. We downloaded the gene sets of c2. cp. KEGG. v6.2.—symbols from the MSigDB database for GSVA analysis after correction. When the corrected *p* value was less than 0.05, it was considered statistically significant. The clusterProfiler R package was used to annotate the functions of m6A-related genes, and the critical value of false discovery rate (FDR) was <0.05.

### Identification of Differentially Expressed Genes Among m6A Patterns

To identify genes associated with m6A modification patterns, patients were divided into different m6A clusters based on the expression levels of 23 m6A regulators. Then, the empirical Bayesian method of the limma R package was used to identify DEGs in three different m6A modification patterns (Ritchie et al., 2015). The significance standard of DEGs was |logFC|> 1, *p* < 0.05.

### Comparison of Tumor Microenvironment Cell Infiltration Among m6A Patterns

To understand the degree of immune cell infiltration in the three subgroups, we quantified the relative abundance of each cell infiltration in the TME of the BC samples using single-sample gene-set enrichment analysis (ssGSEA). Then, we obtained the gene sets for each type of TME infiltrating immune cells and stored a relatively complete subset of human immune cells, including activated CD8 T cells, natural killer T cells, activated DCs, macrophages, and regulatory T cells (Supplementary Table S2). The relative abundance of different infiltrating cells in the TME in each sample was evaluated using the enrichment scores calculated by ssGSEA analysis.

### Construction of m6A Gene Signature

In this study, we applied a method to quantify the m6A modification pattern of individual BC patients. Thus, we constructed a scoring system to evaluate the m6A modification pattern of BC patients, namely, m6Ascore. The specific procedures were as follows.

Firstly, the overlapping DEGs in different m6Aclusters were extracted from all BC samples. These overlapping DEGs were analyzed using unsupervised clustering, and patients were divided into several groups for further analysis. The consistency clustering algorithm was used to determine the number and stability of gene clustering. Next, we used the univariate Cox regression model to analyze the prognosis of each overlapping DEG and selected genes with significant prognostic differences for principal component analysis (PCA) to construct the m6A gene signature. Principal component 1 and principal component 2 were selected as signature scores, and the scores were concentrated on the set of gene blocks with the greatest correlation. In contrast, the contributions of other genes unrelated to other set members were weighted. Finally, m6Ascore was determined in a similar way to the Genome Grading Index (GGI) (Sotiriou et al., 2006; Zhang et al., 2020):

$$m6Ascore=\sum \left(PC1_{i}+PC2_{i}\right),$$

where *i* is the expression of overlapping DEGs with a significant difference in prognosis among m6Aclusters.

### Correlation Between m6A Patterns and Other Relevant Biological Processes

We used the gene set to assess the association between the m6Ascore and important biological processes (Şenbabaoğlu et al., 2016; Zeng et al., 2019), including 1) epithelial-mesenchymal transition (EMT) markers, EMT1, EMT2, and EMT3; 2) immune checkpoint; 3) CD8 T-effector signature; 4) mismatch repair; 5) antigen processing and presentation; 6) antigen processing machinery (APM); 7) Wnt targets; 8) DNA damage repair; 9) nucleotide excision repair; 10) pan-fibroblast transforming growth factor-β response signature (Pan-FTBRS); 11) DNA replication; and 12) angiogenesis signature.

### Statistical Analysis

The Kruskal–Wallis test and one-way ANOVA were used to evaluate the statistical differences among three or more groups (Mariathasan et al., 2018). Spearman’s correlation analysis was used to calculate the correlation coefficient between the expression of m6A regulators and TME infiltrating immune cells. Based on the correlation between the m6Ascore and the patient prognosis, the optimal cut-off point of the data subgroup was determined by the survminer R software package, and the patients were divided into high and low m6Ascore. The Kaplan–Meier method was used to plot the survival curve for the prognosis analysis. The log-rank test was used to determine the statistical significance of the difference. The multivariate Cox regression analysis was used to evaluate the independent prognostic factors. The mutation landscape of the high and low m6Ascore group was presented by the waterfall function of the R software maftools package (Hazra and Gogtay, 2016). The RCircos R package was used to describe the CNV of 23 m6A regulators in 23 pairs of chromosomes. In this study, all tests were bilateral, and *p* < 0.05 was considered statistically significant. All data processing was carried out in R software V3.6.3.

## Results

### The Genetic Variation Landscape of m6A Regulators in Breast Cancer

**FIGURE 1:**
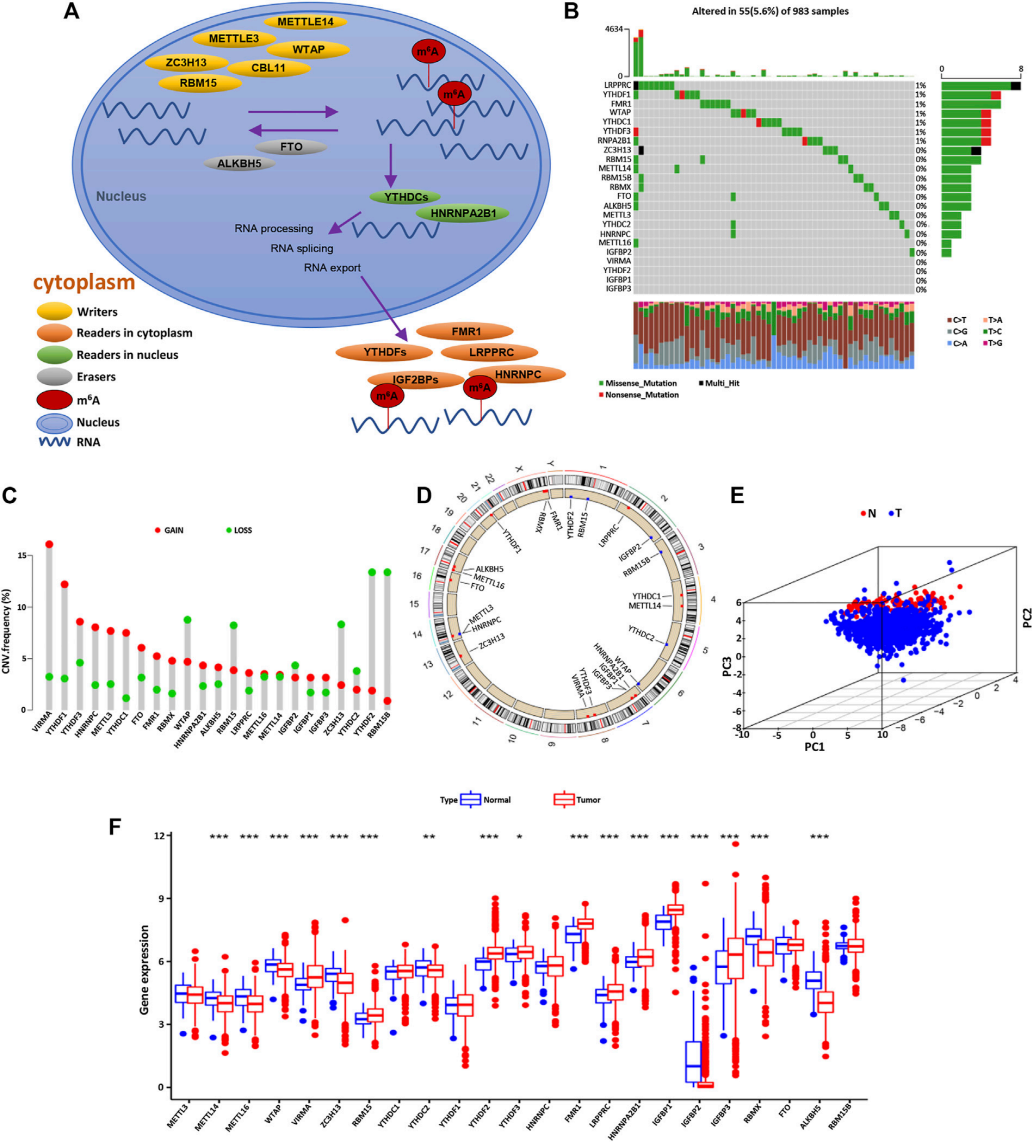
The molecular characteristics and expression variation landscape of m6A regulators in BC. **(A)** Summary of the biological process and potential biological functions of the m6A methylation modified by regulators (writers, erasers, and readers). **(B)** Mutation frequencies of 23 m6A regulators in 983 patients with BC in the TCGA database. Each column represents a single patient, the bar chart above represents TMB, and the number on the right represents the mutation frequency of each regulator. The bar chart on the right shows the proportion of various types of each regulator. The stacked bar chart below shows the percentage of conversion in each sample. **(C)** The CNV frequency of m6A regulators in BC. The green dot represents the deletion frequency, the red dot represents the amplification frequency, and the height of the column represents the change frequency. **(D)** The position of the m6A regulator CNV on 23 chromosomes. **(E)** PCA based on the expression profiles of 23 m6A regulators is used to distinguish tumor tissues from normal tissues. The tumor is marked with blue and the normal breast tissue with red. **(F)** The expression of 23 m6A regulators in normal breast tissue (blue) and tumor tissue (red). The top and bottom of the box represent the quartile range of values. The line in the box represents the median value, and the asterisk above represents the statistical *p* value (*p < 0.05, **p < 0.01, ***p < 0.001).

Firstly, a total of 23 genes that regulate RNA methylation were identified, including eight writers (METTL14, METTL3, RBM15/RBM15B, CBLL1, WTAP, ZC3H13, and VIRMA), two erasers (ALKBH5 and FTO), and 13 readers (FMR1, YTHDF1/2/3, HNRNPA2B1, YTHDC1/2, RBMX, HNRNPC, LRPPPRC, and IGF2BP1/2/3). The dynamic process and mechanism of m6A methylation in RNA modification are described in Figure 1A. Next, we integrated somatic mutation and CNV to explore the mutation rate of 23 m6A regulators in BC. The overall mutation frequency of the m6A regulator was relatively low, and only 55 in 983 samples caused m6A regulator mutation (Figure 1B). Among 23 m6A regulators, LRPPRC, YTHDF1, FMR1, WTAP, YTHDC1, YTHDF3, and RNPA2B1 were mutated in BC, while the other regulatory factors were not mutated. Furthermore, it was found that the CNV alteration frequency of 23 m6A regulators was prevalent in BC, most of which were with the copy number amplification. However, WTAP, RBM15, ZC3H13, YTHDC2, YTHDF2, and RBM15B were with the high frequency of CNV deletion (Figure 1C). The CNV alteration location of the m6A regulators on the chromosome is presented in Figure 1D. Based on the expression levels of these 23 m6A methylation-related regulators, BC samples could be markedly distinguished from normal samples (Figure 1E). Then, we investigated the mRNA expression levels of m6A regulators in BC and normal tissues to determine whether the abnormal expressions were associated with BC malignancy. Compared with normal breast tissue, 17 of the 23 m6A regulators were differentially expressed (Figures 1C,F). These results indicated that the mRNA expression levels of m6A regulators were highly heterogeneous in BC and normal tissues, demonstrating the underlying roles of the abnormal expression pattern of m6A regulators in the oncogenesis and progress of BC.

### m6A Methylation Modification Patterns Mediated by 23 Regulators

**FIGURE 2:**
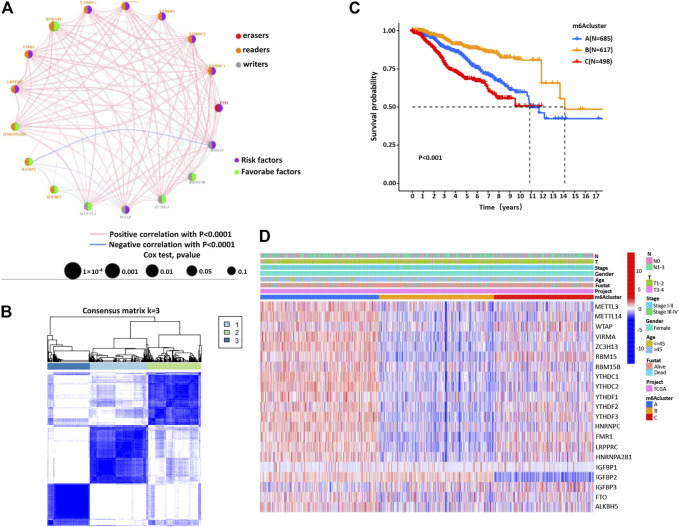
The m6A methylation patterns and their biological characteristics. **(A)** The interaction between m6A regulators in BC. The size of the circle represents the influence of each regulator on the BC prognosis. Orange for readers, gray for writers, and red for erasers. Green dots represent favorable prognostic factors and purple dots represent risk factors. The spectral lines connecting the m6A regulators represent the interaction between them, and the thickness represents the correlation strength between the regulatory factors. The pink line is a positive correlation, and the blue line is a negative correlation. **(B)** Consensus clustering matrix for *k* = 3. **(C)** Kaplan–Meier curves of OS for three m6Acluster in BC patients. **(D)** Unsupervised clustering of 23 m6A regulators in BC patients. m6Acluster, lymph node stage, T stage, tumor stage, survival status, and age are used as the annotation of BC patients. Red represents high expression and blue represents low expression.

Next, the clinical significance of the m6A regulators in BC patients was explored. The network described the interaction between the m6A regulators and their impact on the BC prognosis (Figure 2A, Supplementary Table S3). This result denoted that some m6A regulators (such as YTHDF1 and FTO) were associated with a poor prognosis. However, other regulatory factors (such as HNRNPC and IGFBR3) were associated with a good prognosis of BC patients (Supplementary Figures S2–S4). Moreover, the expression of m6A regulators of the same functional category also showed a significant correlation. We then classified the patients with different m6A modification patterns based on the expression levels of 23 m6A regulators. By model-based cluster analysis, three different methylation modification patterns were identified, including 685 cases in m6ACluster A, 617 cases in m6ACluster B, and 498 cases in m6ACluster C (Figure 2B, Supplementary Table S4). Prognostic analysis of three major subtypes of m6A modification showed that the m6ACluster B modification pattern had a particularly significant survival advantage (Figure 2C). Moreover, the relationship between clinical factors and gene expression of three different m6A methylation patterns was analyzed and presented in Figure 2D.

**FIGURE 3:**
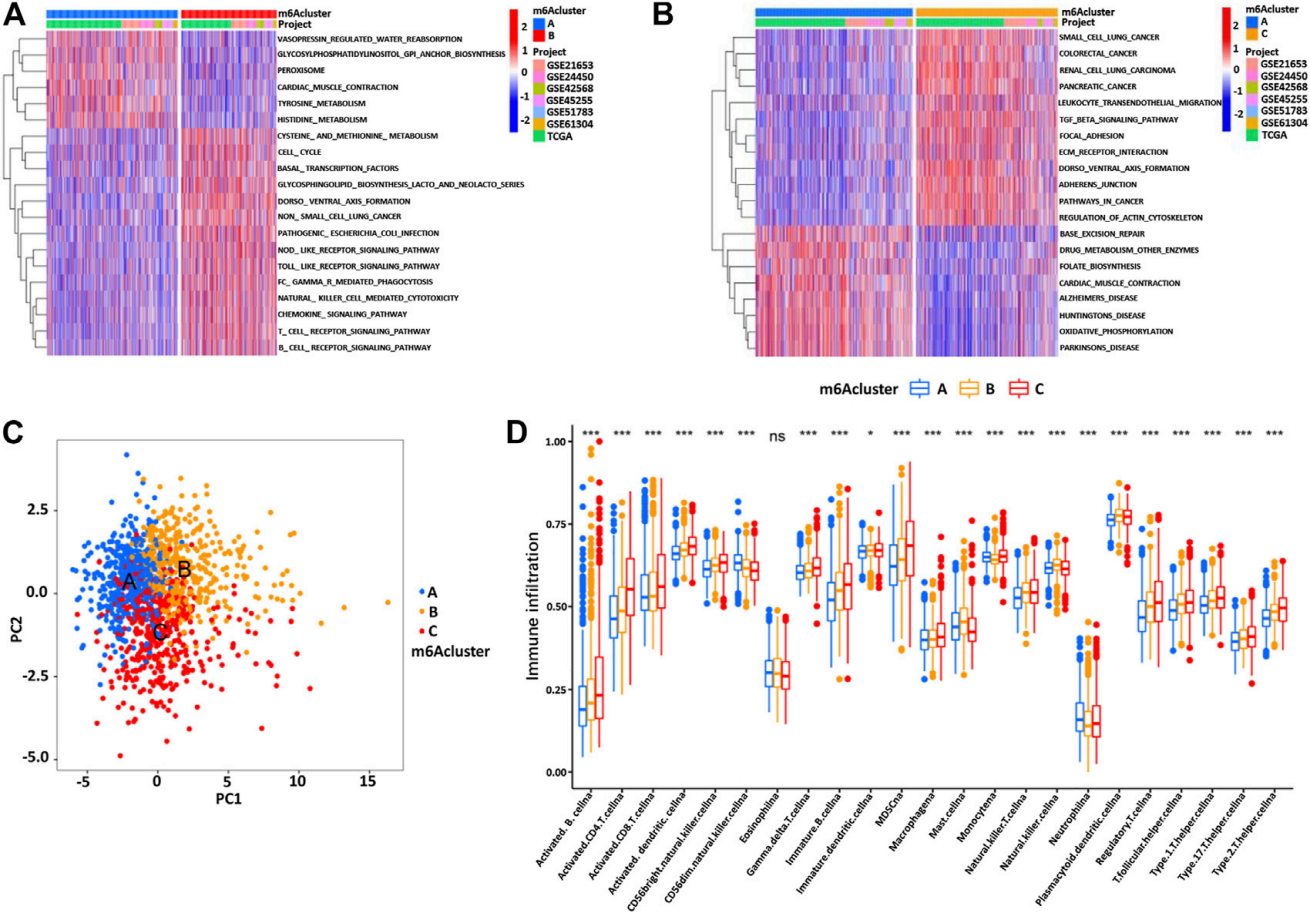
**(A,B)** The GSVA enrichment analysis of biological pathway activation under different m6Aclusters. Red represents the activation pathway and blue represents the inhibition pathway. **(A)** m6Acluster A compared with m6Acluster B. **(B)** m6Acluster A compared with m6Acluster C. **(C)** PCA shows significant differences in the transcriptome profiles of three m6Aclusters. **(D)** The expression abundance of different TME infiltrating cells in three m6Aclusters. The upper and lower end of the box represents the quartile range of the value, the middle line represents the median value, and the asterisk represents the statistical *p* value (*p < 0.05, **p < 0.01, ***p < 0.001).

Through GSVA enrichment analysis, we then intended to investigate the enrichment of biological processes in these different m6A modification patterns. m6ACluster A was significantly correlated with immunosuppression and other biological processes. m6ACluster B was significantly enriched in the pathways related to complete immune activation, including Toll_like_receptor_signaling_pathway, T_cell_receptor_signaling_pathway, B_cell_receptor_signaling_pathway, and Chemokine_signaling_pathway. Moreover, m6Acluster C showed significant enrichment in carcinogenic activation and matrix pathway, such as TGF_beta_signaling_pathway, Adherens_junction, and ECM_receptor_interaction (Figures 3A,B, Supplementary Table S5). PCA was used to analyze the transcriptome profiles of the three m6A modification patterns, showing significant differences in the transcriptome profiles of different modification patterns (Figure 3C). In the subsequent analysis of TME cell infiltration, it was found that the m6ACluster C was enriched in the infiltration of innate immune cells, such as macrophages, myeloid-derived suppressor cells, natural killer cells, monocytes, regulatory T cells, CD8 T cells, and DCs (Figure 3D, Supplementary Table S6). However, according to the results of our survival analysis, the patients with m6ACluster C did not possess the corresponding survival advantages. It has been proven that tumors with immune rejection phenotype were characterized by the presence of a large number of immune cells, which stayed in the matrix surrounding the tumor cell nest and did not penetrate the central zone (Zhang et al., 2020).

### Functional Annotation of m6A Methylation Modification Patterns

**FIGURE 4:**
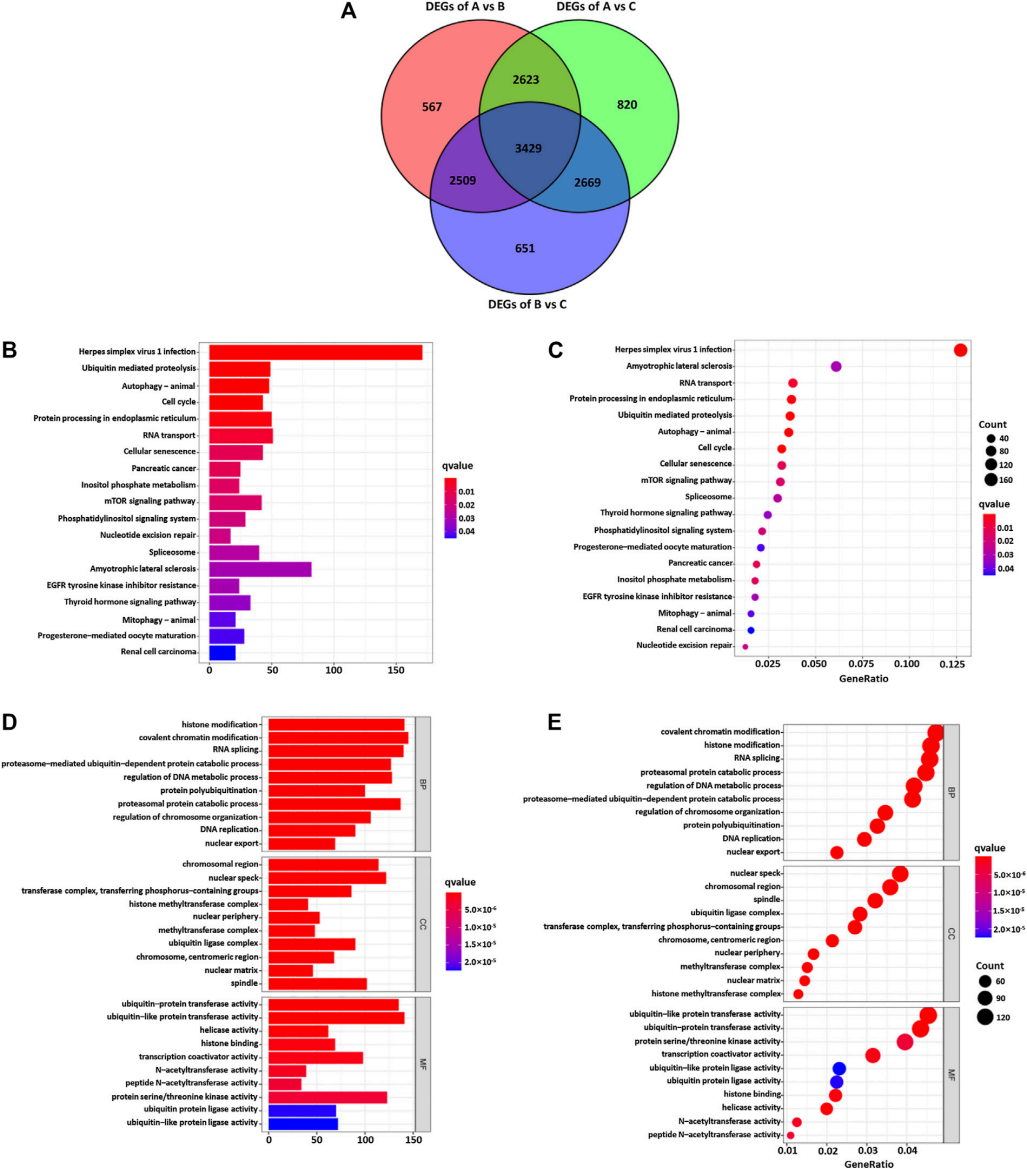
**(A)** 3426 m6A subtype-related genes presented in the Venn diagram. The KEGG enrichment analysis **(B,C)** and GO functional annotation **(D,E)** are performed for the m6A related genes.

In order to explore the potential biological regulatory pathways in the different m6A modification patterns, we successfully identified 3429 DEGs associated with the m6A phenotype (Figure 4A). The clusterProfiler software package was used to analyze the Gene Ontology (GO) and Kyoto Encyclopedia of Genes and Genomes (KEGG) function enrichment of the DEGs. The results are shown in Figures 4B–E. The KEGG analysis showed that the DEGs were enriched in the cell cycle, EGFR tyrosine kinase inhibitor resistance, and mTOR signaling pathway (Figures 4B,C). The GO analysis of the biological process showed that these DEGs were enriched in DNA replication, regulation of DNA metabolism, and histone modification (Figures 4D,E). Furthermore, the cellular component analysis showed that DEGs were abundant in histone methyltransferase complex and transferase complex and transferring phosphorus-containing groups. Molecular function analysis indicated that DEGs were mainly located in the helicase activity and transcription coactivator activity (Figures 4D,E).

### Construction of m6A Gene Signature and Functional Annotation

**FIGURE 5:**
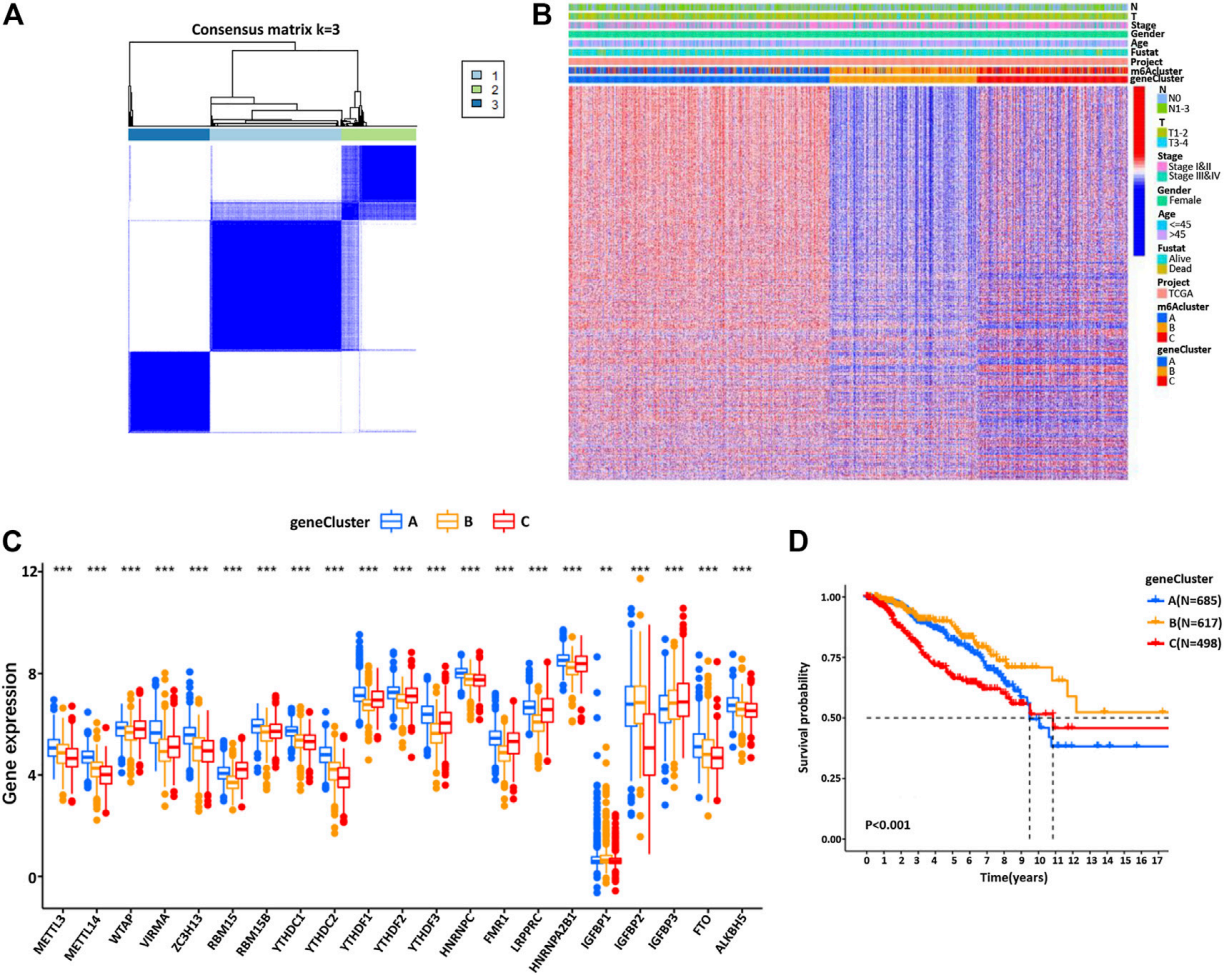
**(A)** Pearson correlation analysis was used to explore the relationship between the m6A regulators, and the common clustering matrix of *k* = 3 was selected. **(B)** In the BC cohort, the unsupervised clustering analysis of the m6A phenotype-related genes overlapped and divided the patients into different genomic subtypes, named m6A gene clusters A, B, and C, respectively. Gene clusters, m6Aclusters, BC stage, T stage, lymph node metastasis stage, survival status, and patient age were annotated. **(C)** The expression of 23 m6A regulators in three gene clusters. The top and bottom of the box represent the quartile range. The middle line represents the median value, and the asterisk represents the statistical *p* value (*p < 0.05, **p < 0.01, ***p < 0.001). The differences among the three gene clusters are tested by one-way ANOVA. **(D)** Kaplan–Meier survival curve showed that m6A gene-modified phenotype was correlated with the OS rate in BC patients.

To further understand this regulatory mechanism, we divided the patients into different genotypes by unsupervised cluster analysis of the 3429 genes related to the phenotype of m6A, using the R limma package from the transcriptomic profile of TCGA and GEO. By model-based cluster analysis, we finally identified three different methylation modification patterns, including 685 cases in gene cluster A, 617 cases in gene cluster B, and 498 cases in gene cluster C (Figure 5A). The results were consistent with the cluster grouping of m6A modification patterns, revealing three different m6A modified genomic phenotypes, named m6A gene cluster A, cluster B, and cluster C (Supplementary Figures S4A–D, Figure 5B, Supplementary Table S4). This indicates that these three different methylation patterns of m6A did exist in BC. There were significant differences in the expression of m6A regulators in the three m6A gene clusters, which was consistent with the expected results of previous methylation modification patterns of m6A (Figure 5C). Furthermore, there was an observable good prognosis in gene cluster B and a poor prognosis in gene cluster C (Figure 5D). However, the above could only be based on the analysis of the patient population and could not accurately predict the methylation pattern of m6A in individual patients.

**FIGURE 6:**
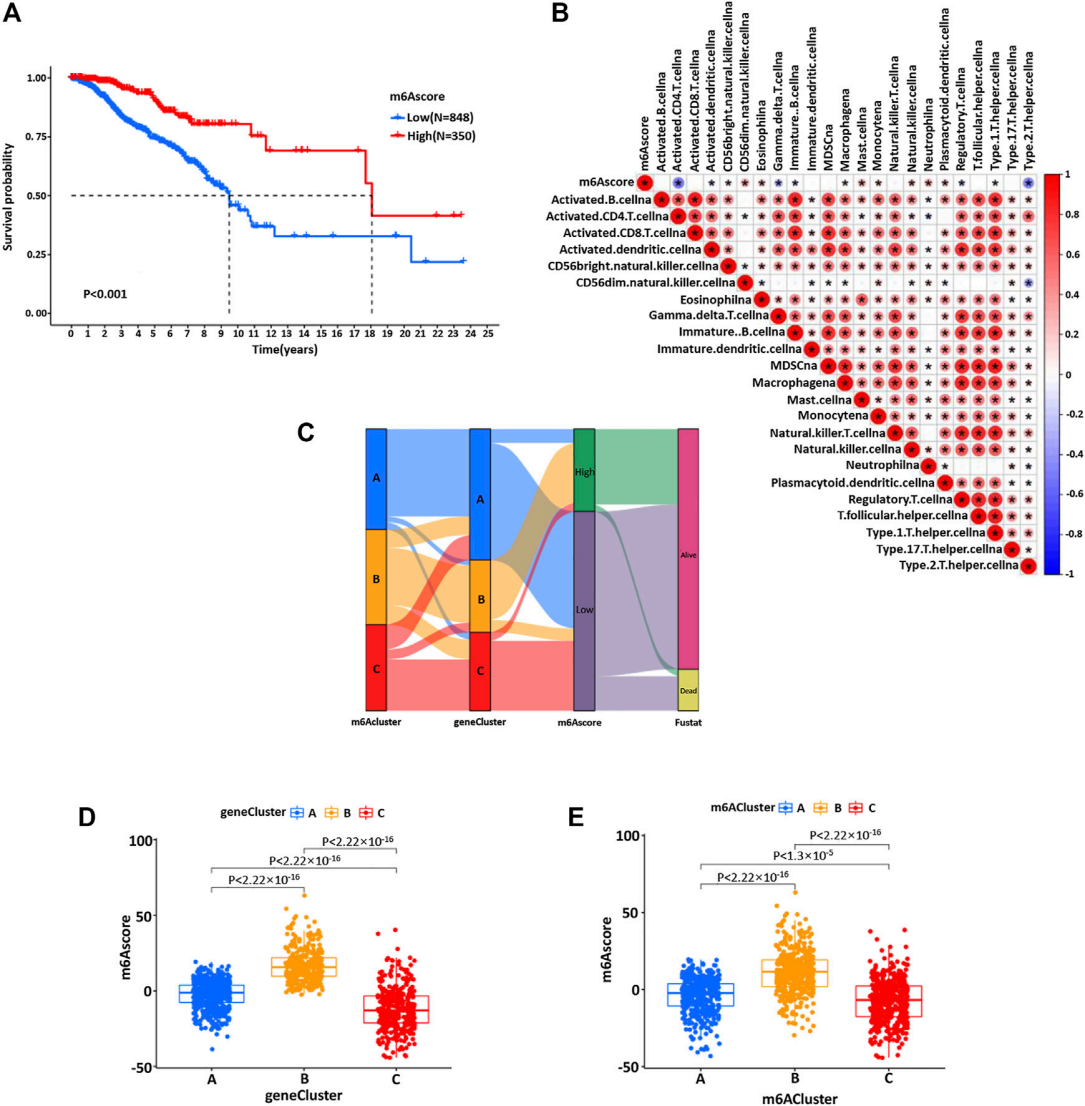
**(A)** Kaplan–Meier curve was used to analyze the survival rate of the patients with high and low m6Ascores in the BC cohort (*p* < 0.001). **(B)** Spearman was used to analyze the correlation between m6Ascore and genetic characteristics in the BC cohort. Red represents positive correlation and blue represents negative correlation. **(C)** The alluvial map showed the changes of m6Acluster, gene cluster, m6Ascore, and survival status. **(D)** Kruskal–Wallis test was used to compare the statistical difference between three gene clusters in the BC cohort (*p* < 0.001). **(E)** The difference of the scores of three m6Aclusters in the BC cohort (*p* < 0.001, Kruskal–Wallis test).

Due to the individual differences and complexity of m6A methylation modification, we constructed a set of m6A modification models that could quantify the individual BC, namely, the m6Ascore. The changes in individual patient attributes are shown by the alluvial chart (Figure 6C). The optimal cut-off value of 7.795803 was calculated using the survminer software package, and patients were successfully divided into a high group and low group according to m6Ascore (Figure 6A). These results indicated that patients with a high score of the m6A group had a significant survival benefit. Then, the correlation between the m6Ascore and biological process was analyzed to clarify the genetic characteristics of m6A (Figure 6B). Besides, the Kruskal–Wallis test revealed a significant difference in m6Ascore among m6A gene clusters (Figure 6D). Gene cluster C showed the lowest median score, while gene cluster B showed the highest median score, indicating that the low m6Ascore group might be closely related to immune activation-related signatures, whereas the high m6Ascore group could be related to stromal activation-related signatures (Figure 6D). The score of m6Acluster B was higher than that of m6Aclusters A and C, and the m6Ascore of m6Acluster C was the lowest (Figure 6E).

### Characteristics of m6A Modification in Tumor Somatic Mutation

**FIGURE 7:**
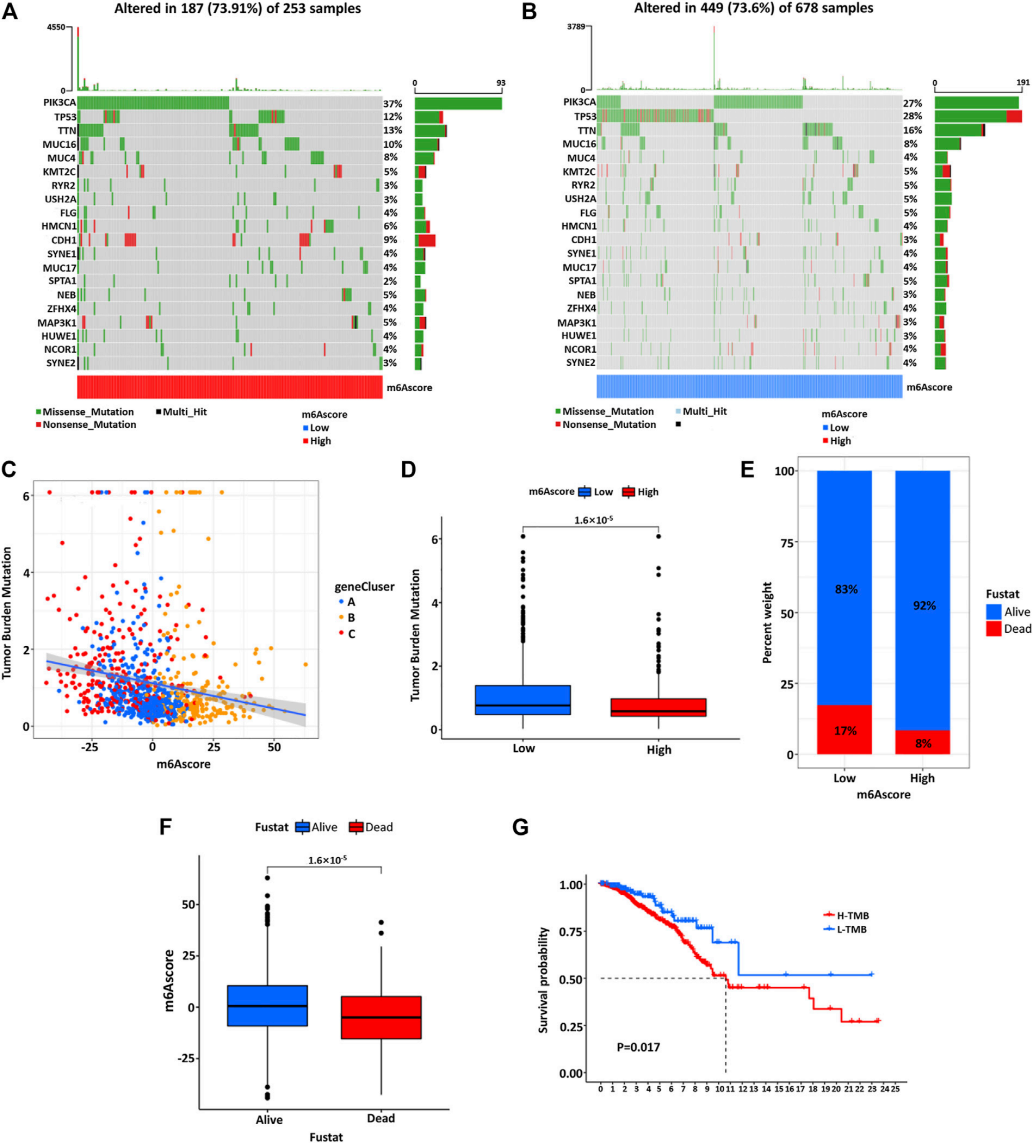
The waterfall diagram of tumor somatic mutation in patients with high **(A)** and low **(B)** m6Ascore. Each column represents a single patient, the bar chart above represents TMB, and the number on the right shows the mutation frequency of each gene. The bar chart on the right shows the proportion of each variation type. **(C)** There is a significant negative correlation between the m6Ascore and TMB. **(D)** Quantitative analysis of TMB shows a significant negative correlation between the m6Ascore of tumors and TMB. **(E)** The proportion of patients with different survival statuses in low or high m6Ascore groups. **(F)** The difference of m6Ascore in different survival status groups. **(G)** Kaplan–Meier curve was used to analyze the survival of the high and low TMB load.

Then, we used the maftools software package to analyze the distribution of somatic mutations between high and low m6Ascore of BC patients in the TCGA database (Figures 7A,B). The results showed that the mutation rate of PIK3CA was relatively high in the high m6Ascore cohort compared to the low m6Ascore cohort (37 *vs*. 27%). Subsequently, quantitative analysis of tumor mutational burden (TMB) confirmed that the m6Ascore was significantly negatively correlated with TMB (Figure 7C). The patients with low m6Ascore were significantly associated with the higher TMB (Figure 7D). At the same time, the m6Ascore analysis showed that the low M6Score group had a higher proportion of patient death (Figure 7E) and the average m6Ascore was higher in alive patients than dead patients (Figure 7F). Survival analysis of patients showed that patients with low TMB had a more significant prognostic advantage than high TMB patients (Figure 7G). To predict the molecular subtypes of BC samples from TCGA, we explored the proportion of basal, her2, luminal A, luminal B, and normal patients in the low and high m6Ascore groups. It was found that the luminal A type accounted for a large proportion in the high m6Ascore group, while the basal type accounted for a large proportion in the low m6Ascore group (Supplementary Figure S6A). Then, according to our established score, the basal subtype significantly exhibited the lowest m6Ascore among these subtypes, consistent with the overall prognosis of BC patients (Supplementary Figure S6B). The above results were consistent with the previous conclusion (Figure 6A).

### The Role of m6A Modification Pattern in Anti-PD-1/L1 Immunotherapy

**FIGURE 8:**
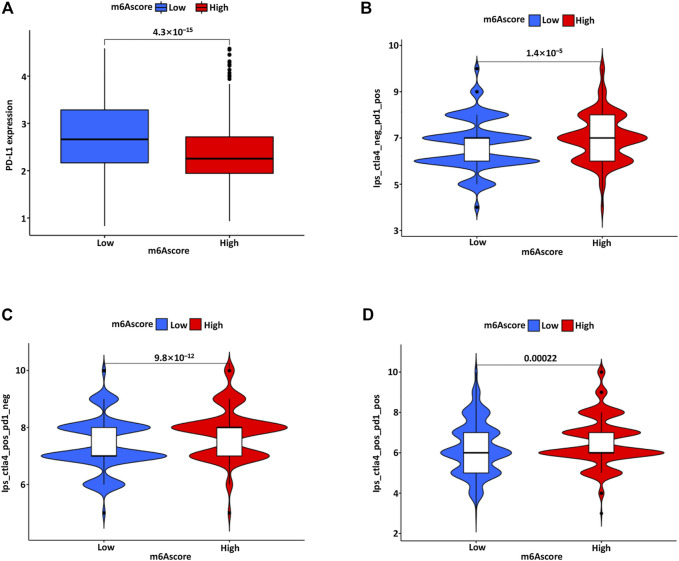
**(A)** The PD-L1 expression in the low and high m6Ascore group. The association between IPS and m6Ascore model, including IPS-PD1 **(B)**, IPS-CTLA4 **(C)**, and IPS-PD1/CTLA4 **(D)** scores in the low and high m6Ascore group.

Clinically, the PD-L1 expression of immune cells was considered separately for PD-L1 immune therapy, and the immune cell proportion score (IPS) was introduced as an indicator to distinguish beneficiaries. Furthermore, we also explored the association between IPS and the m6Ascore in BC. The IPS-PD1, IPS-CTLA4, and IPS-PD1/CTLA4 scores were significantly increased in the high m6Ascore group (Figures 8A–D). The treatment benefits from antibodies against immune checkpoints of PD-1 and CTLA-4 might differ between the high and low m6Ascore groups. There is evidence that patients with high TMB status have a relatively durable clinical response to PD-1/PD-L1 immunotherapy (Luchini et al., 2019). The above analyses proved that the m6A modification mode is intensively associated with TMB and PD-1/PD-L1 immunotherapy.

## Discussion

As an important epigenetic modification, m6A methylation is identified as the most popular RNA modification in eukaryotes. M6A modification is critical in immune response, inflammation, tumorigenesis, and drug resistance. Robust evidence has confirmed the dysregulated expression patterns of m6A in tumor development and progression, but their association with immune infiltration in TME of BC remains obscure. The main purpose of this study is to construct the m6Ascore based on 23 m6A methylation modification patterns and validate its correlation with the tumor immune microenvironment in BC. Specifically, our analysis further confirmed that m6Ascore was correlated with immunotherapy, represented by anti-PD-1/PD-L1 immunotherapy.

At present, the expression patterns of estrogen receptor (ER), progesterone receptor (PR), and human epidermal growth factor receptor type 2 (HER2) in different subtypes of BC represent a predictive method for the therapeutic guidance of BC. However, the existing classification model based on these molecules cannot accurately reflect the tumor heterogeneity and evaluate the prognosis of BC. In this study, we successfully identified three patterns of m6A methylation, named m6Acluster A, m6Acluster B, and m6Acluster C, based on 23 regulatory factors associated with m6A methylation in different immune environments. These patterns had distinct infiltrating characteristics of TME cells. m6Acluster A presented an immune desert phenotype and immunosuppression, while m6Acluster B presented an immune inflammation phenotype characterized by immune activation. The phenotype of m6Acluster C was immune rejection, mainly manifested by interstitial activation and innate immune cells infiltration. Immune rejection and immune desert types are considered as non-inflammatory tumors and may exhibit extensive immune cell infiltration in TME (Turley et al., 2015; Chen and Mellman, 2017; Mayakonda et al., 2018). Notably, in the immune rejection phenotype, many immune cells remain in the stroma around the tumor cell nests and do not penetrate the tumor parenchyma. The immune desert phenotype is associated with a lack of activating and initiating T cells, immune tolerance, and ignorance (Salmon et al., 2012). Our analysis confirmed that m6Acluster C showed significant oncogenic activation and enrichment of matrix pathways, including TGF-β pathways, adhesion junctions, and ECM receptor interactions, which were significantly associated with T-cell inhibition. Our results also showed that the comprehensive analysis of the infiltrating characteristics of TME cells established by different m6A modification methods showed that m6Acluster C activated innate immunity but had a poor prognosis. Therefore, the reliability of our classification of different m6A modification modes was verified, according to the invasion characteristics of TME cells in each cluster. In addition, we also analyzed the differentially expressed mRNA in different m6Acluster. The results indicated that these differentially expressed mRNAs were important m6A related genes in BC and were significantly correlated with the biological and immune-immune-related pathways of m6A modification. Subsequently, we also identified three different genomic subtypes based on the m6A-related genes, similar to the previous clustering results of the m6A-modified phenotypes. These genomic subtypes were also significantly associated with immune activation. This result highlights the significant roles of m6A modification in presenting different TME landscapes. In our scoring system, the m6A modification pattern characterized by immune rejection phenotype corresponded to a higher m6Ascore, while the m6Ascore characterized by immune inflammation phenotype was relatively lower. This m6A scoring system is a reliable and comprehensive evaluation system, that can be used to evaluate the m6A modification mode and determine the infiltrating characteristics of TME cells in BC.

Emerging studies have deciphered the relationship between infiltrating immune TME and m6A modification. For example, Li et al. established a valuable m6Ascore system based on 22 m6A regulators (Kim and Chen, 2016). The m6A modification patterns were capable of predicting the tumor immune microenvironment and the prognosis of HCC. Zhang et al. also constructed the m6Ascore to quantify its potential in gastric cancer (Zhang et al., 2020). They confirmed that m6A modification was critical in shaping the diversity and complexity of TME and that this score offered an unambiguous characterization of TME infiltration, thus guiding more effective immunotherapy strategies. In our study, the m6Ascore was negatively correlated with TMB and PD-L1 expression. Methylation patterns played an indispensable role in the formation of the immune TME landscape, suggesting that m6A modification might influence the therapeutic effect of ICB. In addition, previous studies have shown that checkpoint immunotherapy responses are related to not only antigen processing but also angiogenesis inhibition, TGF-β pathway components, and EMT. Herein, we hypothesized that the activated immune microenvironment mediated the resistance to ICB and influenced personalized precision immunotherapy for BC. Integration of the m6A gene with biomarkers such as PD-L1 expression, mutation load, and immune TME, might be an effective prediction method for evaluating immunotherapy.

Nevertheless, there are still some problems to be solved in our study. Firstly, further explorations are needed to verify the accuracy of this signature in predicting the immune state of BC patients. Although we have theoretically proved the value of these m6A regulators, their roles in BC samples are still not yet fully elucidated in this study. The accuracy and credibility of prediction effects will be greatly improved by database analysis in collaboration with real-world prospective clinical sample validation. Secondly, the biological mechanism of specific m6A-related regulators in BC oncogenesis and progression is worthy of further experimental verification. In the end, the combination of risk score, TNM system, and other routine detection methods is necessary for clinical prognosis evaluation. We would like to emphasize that our model is a useful complement to the clinical system, not a replacement.

## Conclusion

In summary, the constructed m6Ascore could comprehensively evaluate individual m6A methylation patterns and corresponding TME cell infiltration characteristics and judge tumor immunophenotype. In addition, we also confirmed the relationship between the m6Ascore and clinicopathological features, which may even help evaluate the clinical efficacy of anti-PD-1/PD-L1 immunotherapy. The illustration of m6A regulatory factors or genes associated with m6A phenotypes will provide novel strategies for personalized evaluation in BC treatment.

## Data Availability

The datasets presented in this study can be found in online repositories. The names of the repository/repositories and accession number(s) can be found in the article/Supplementary Material.

## Abbreviations

BC, breast cancer; CNV, copy number variation; CTLA-4, cytotoxic T-lymphocyte-associated antigen 4; DC, dendritic cells; DEGs, differentially expressed genes; GEO, Gene-Expression Omnibus; GSVA, gene-set variation analysis; GGI, Genome Grading Index; ICB, immune checkpoint blockade; lncRNAs, long non-coding RNAs; m6A, N6-methyladenosine; OS, overall survival; PCA, principal component analysis; PD-1/PD-L1, programmed death; ssGSEA, single-sample gene-set enrichment analysis; TCGA, The Cancer Genome Atlas; TCGA-BC, The Cancer Genome Atlas-BC; TME, tumor microenvironment; TMB, tumor mutational burden; HCC, hepatocellular carcinoma; CAF, cancer-related fibroblasts; GDC, Genomic Data Commons; EMT, epithelial-mesenchymal transition; IPS, immune cell proportion score; KEGG, Kyoto Encyclopedia of Genes and Genomes; FDR, false discovery rate; GO, Gene Ontology; ER, estrogen receptor; PR, progesterone receptor; HER2, human epidermal growth factor receptor type 2.
