# Supplementary figures and images for: Analysis of m6A Methylation Modification Patterns and Tumor Immune Microenvironment in Breast Cancer
Source: Front Cell Dev Biol. 2022 Feb 1;10:785058. doi: 10.3389/fcell.2022.785058 (PMC8846385; doi:10.3389/fcell.2022.785058)

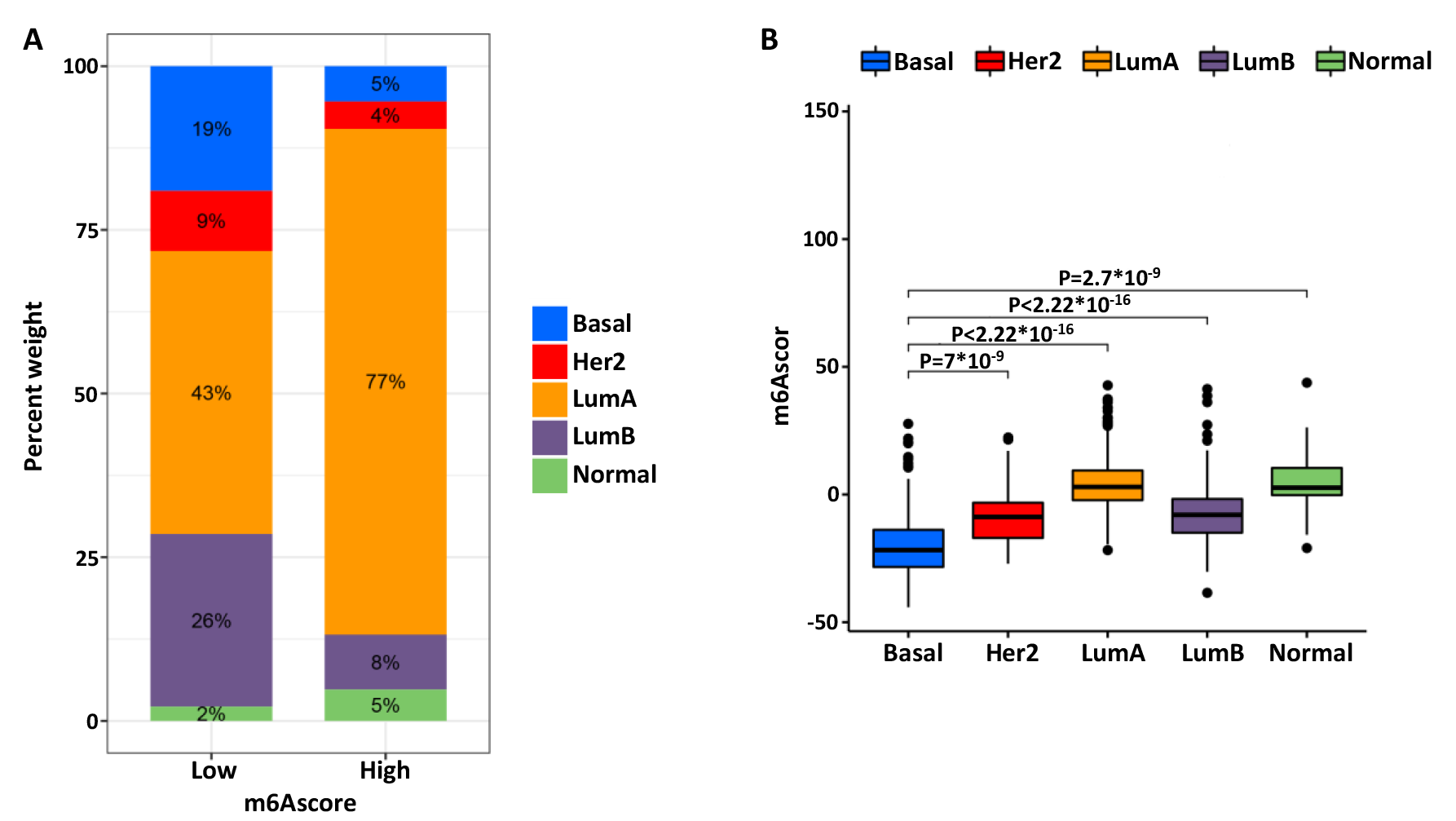

Supplement: Supplementary file 2 [file Image6.TIF]

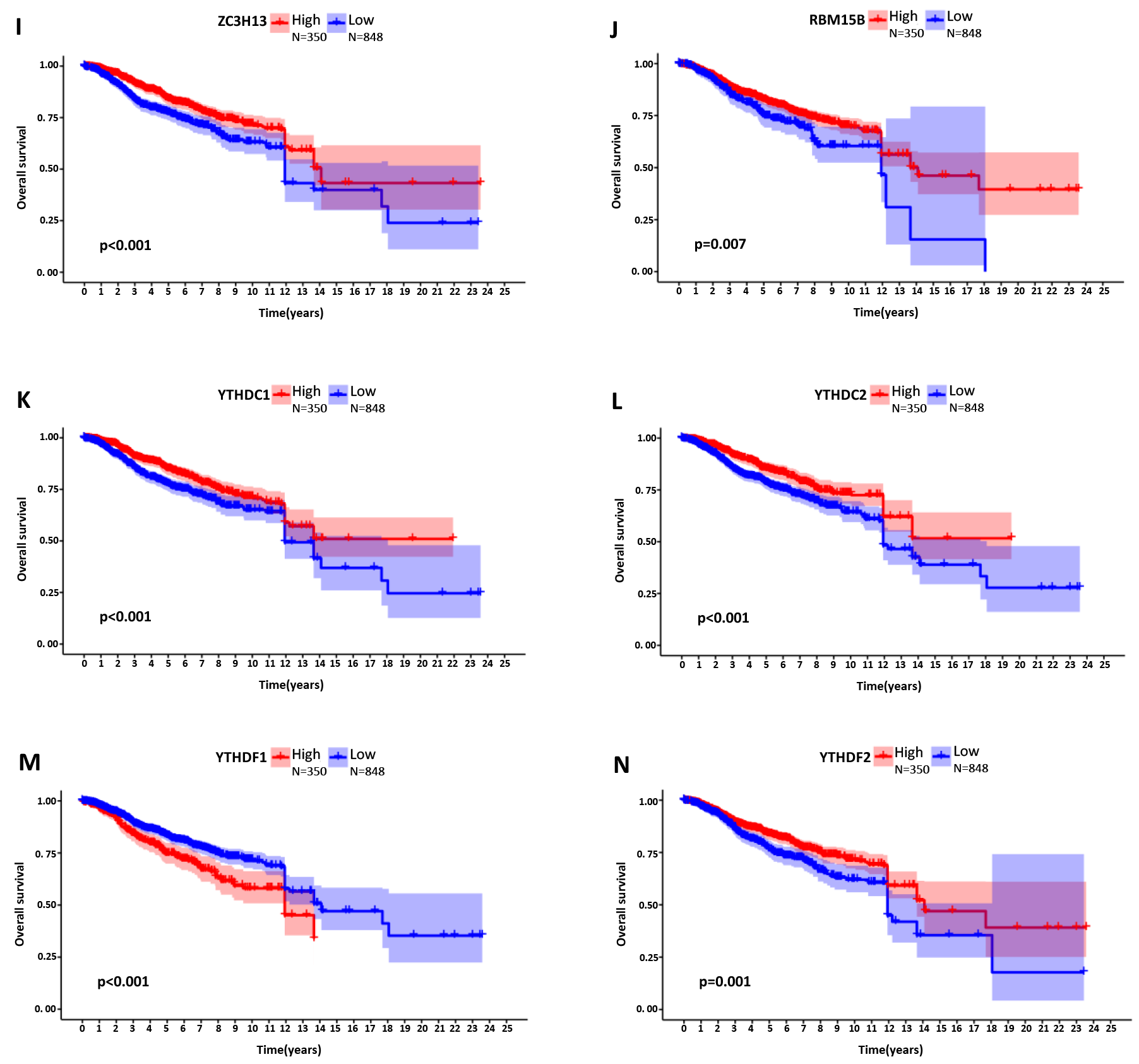

Supplement: Supplementary file 4 [file Image3.TIF]

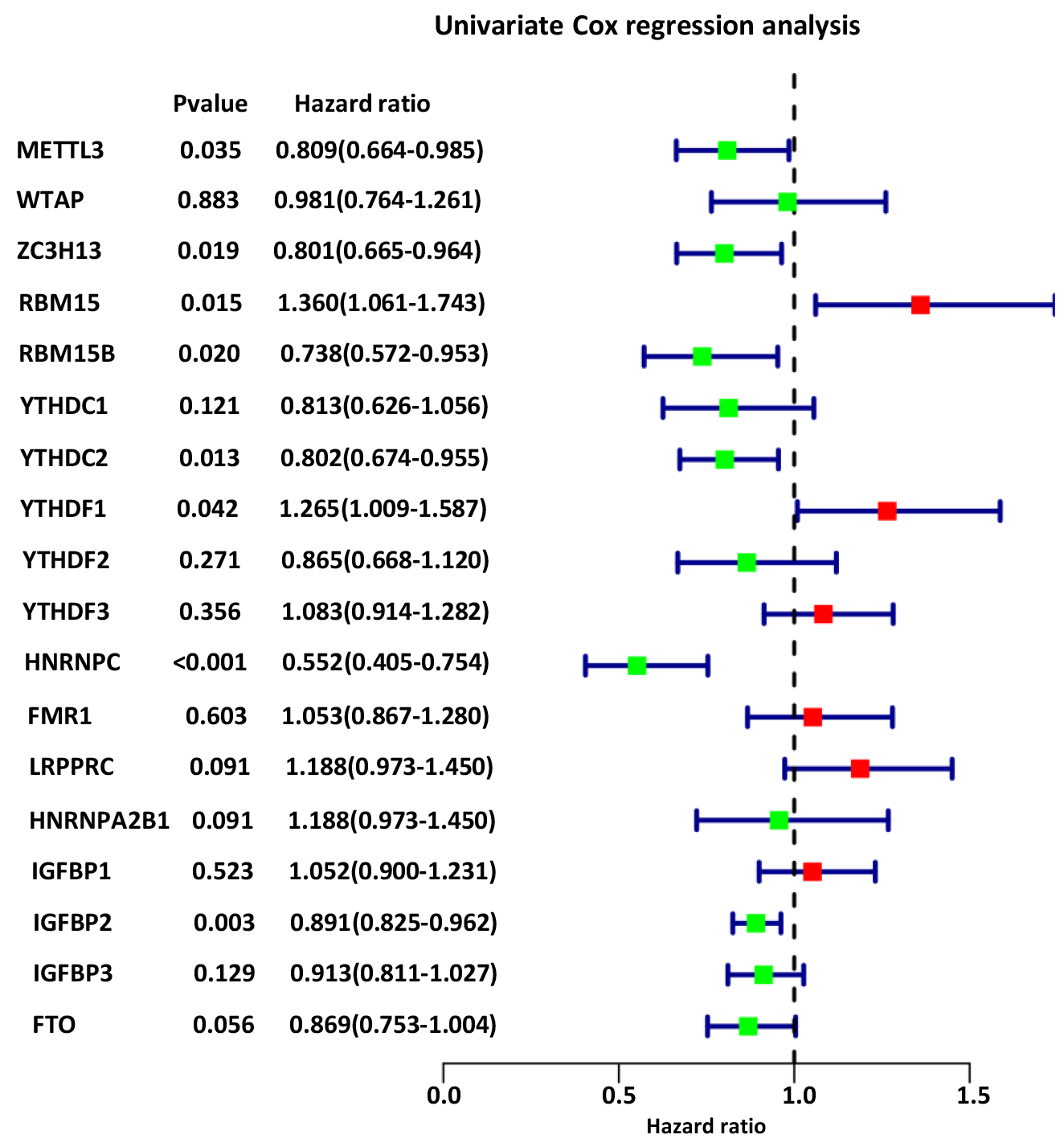

Supplement: Supplementary file 5 [file Image4.TIF]

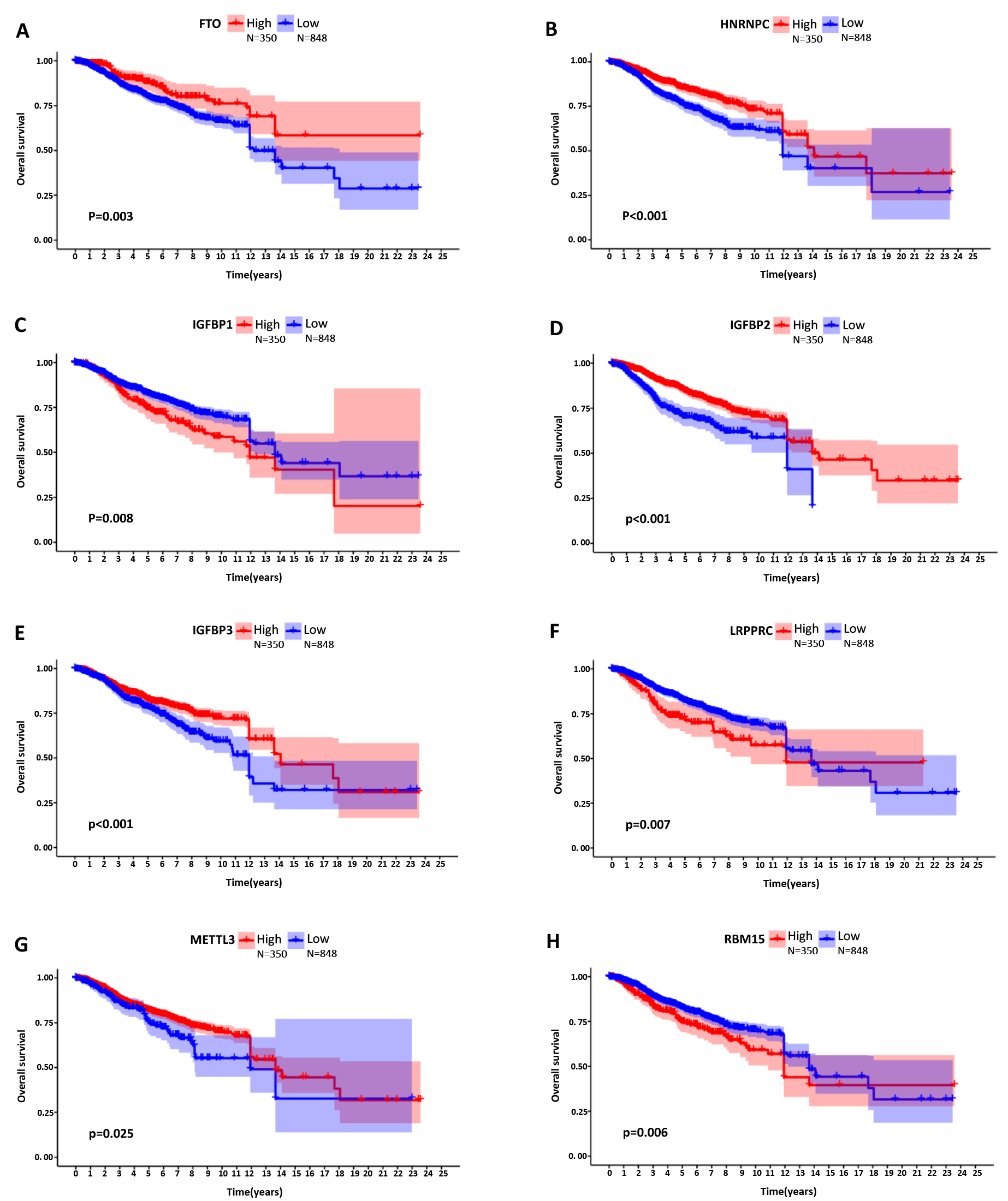

Supplement: Supplementary file 6 [file Image2.TIF]

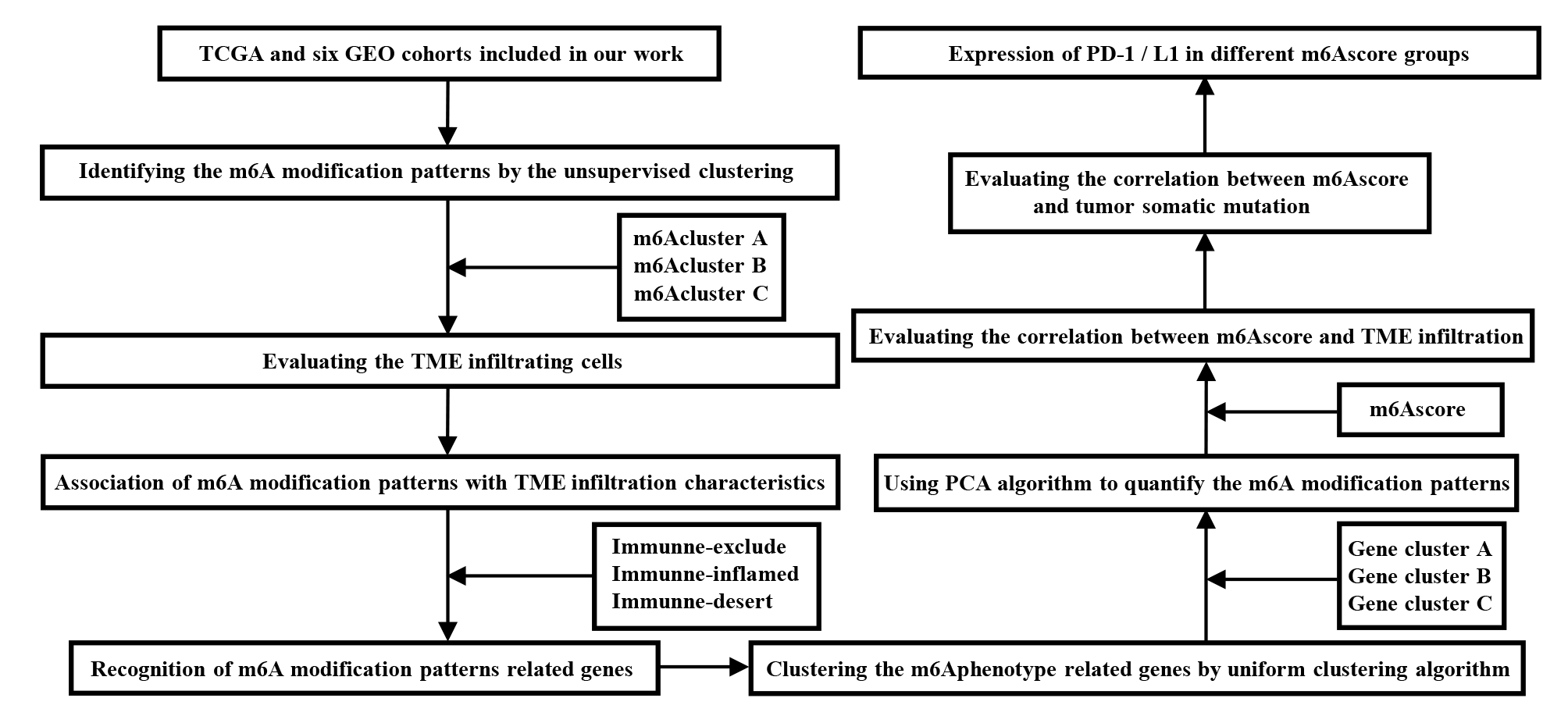

Supplement: Supplementary file 7 [file Image1.TIF]

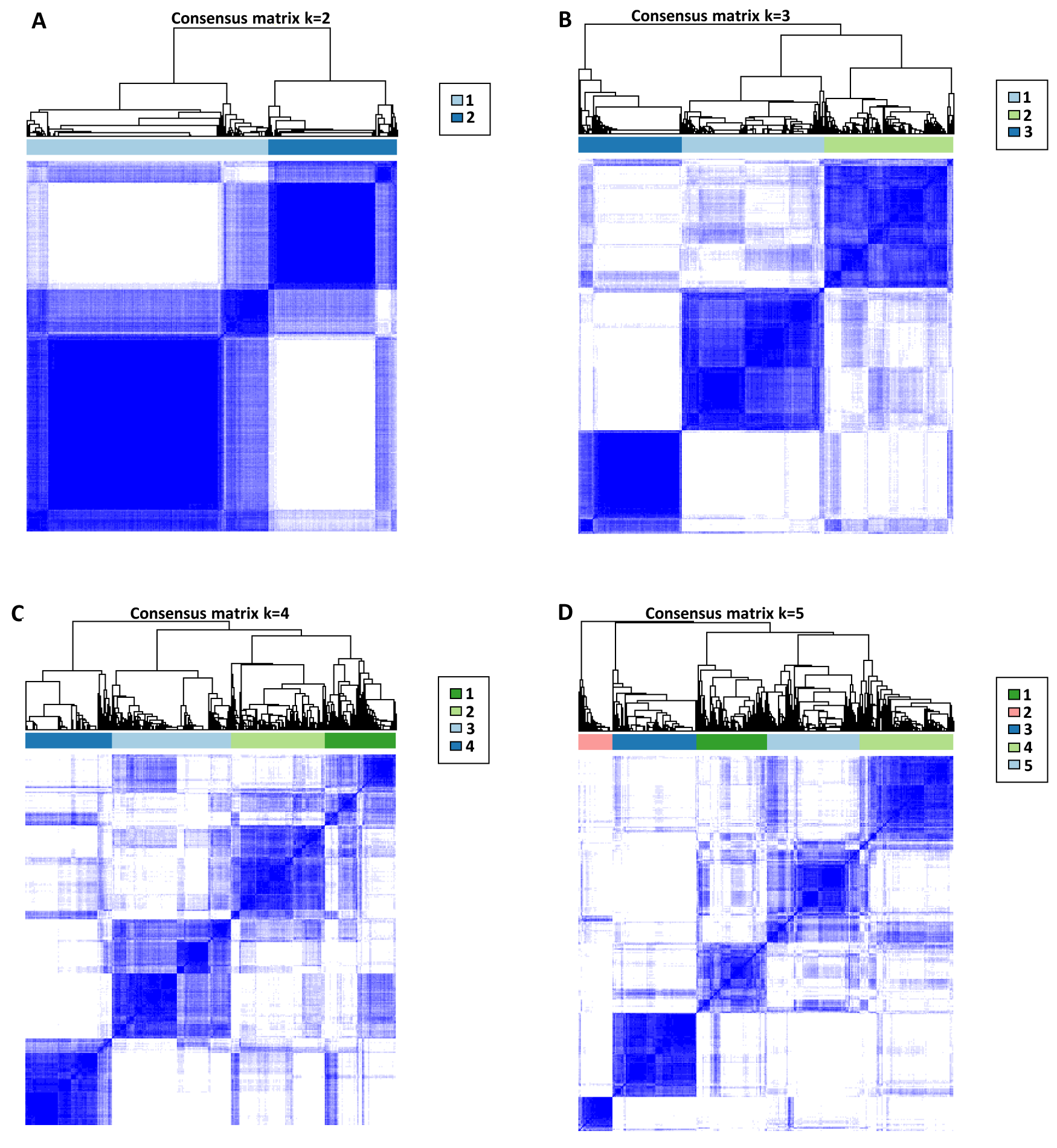

Supplement: Supplementary file 11 [file Image5.TIF]
