# Supplementary material for: Analysis of m6A Methylation Modification Patterns and Tumor Immune Microenvironment in Breast Cancer
Source: Front Cell Dev Biol. 2022 Feb 1;10:785058. doi: 10.3389/fcell.2022.785058 (PMC8846385; doi:10.3389/fcell.2022.785058)
Supplement: Supplementary file 3 [file Table1.DOCX]

**Table S1.** The information of the utilized GEO datasets in our study

| **Data set** | **Topics** | **Number of samples** | **Original study** |
| --- | --- | --- | --- |
| GSE21653 | A gene expression signature identifies two prognostic subgroups of basal breast cancer | 266 breast tumors |  |
| GSE42568 | Breast Cancer Gene Expression Analysis | 104 breast tumors; 17 normal breast |  |
| GSE45255 | Expression Profiles of Breast Tumors from Singapore and Europe | 149 breast tumors |  |
| GSE51783 | Comparison of Poly(A) capture versus Ribosomal RNA depletion methods for RNA-seq | 11 breast tumors |  |
| GSE61304 | Novel bio-marker discovery for stratification and prognosis of breast cancer patients | 58 breast tumors; 4 normal |  |
| GSE24450 | 183 breast tumors from the Helsinki Univerisity Central Hospital with survival information | 183 breast tumors |  |
