# Supplementary material for: Analysis of m6A Methylation Modification Patterns and Tumor Immune Microenvironment in Breast Cancer
Source: Front Cell Dev Biol. 2022 Feb 1;10:785058. doi: 10.3389/fcell.2022.785058 (PMC8846385; doi:10.3389/fcell.2022.785058)
Supplement: Supplementary file 9 [file Table2.docx]

**Table S2: The gene sets for marking each TME infiltration cell type**

| **Metagene** | **Cell.type** | **Immunity** |
| --- | --- | --- |
| ADAM28 | Activated.B.cell | Adaptive |
| CD180 | Activated.B.cell | Adaptive |
| CD79B | Activated.B.cell | Adaptive |
| BLK | Activated.B.cell | Adaptive |
| CD19 | Activated.B.cell | Adaptive |
| MS4A1 | Activated.B.cell | Adaptive |
| TNFRSF17 | Activated.B.cell | Adaptive |
| IGHM | Activated.B.cell | Adaptive |
| GNG7 | Activated.B.cell | Adaptive |
| MICAL3 | Activated.B.cell | Adaptive |
| SPIB | Activated.B.cell | Adaptive |
| HLA-DOB | Activated.B.cell | Adaptive |
| IGKC | Activated.B.cell | Adaptive |
| PNOC | Activated.B.cell | Adaptive |
| FCRL2 | Activated.B.cell | Adaptive |
| BACH2 | Activated.B.cell | Adaptive |
| CR2 | Activated.B.cell | Adaptive |
| TCL1A | Activated.B.cell | Adaptive |
| AKNA | Activated.B.cell | Adaptive |
| ARHGAP25 | Activated.B.cell | Adaptive |
| CCL21 | Activated.B.cell | Adaptive |
| CD27 | Activated.B.cell | Adaptive |
| CD38 | Activated.B.cell | Adaptive |
| CLEC17A | Activated.B.cell | Adaptive |
| CLEC9A | Activated.B.cell | Adaptive |
| CLECL1 | Activated.B.cell | Adaptive |
| AIM2 | Activated.CD4.T.cell | Adaptive |
| BIRC3 | Activated.CD4.T.cell | Adaptive |
| BRIP1 | Activated.CD4.T.cell | Adaptive |
| CCL20 | Activated.CD4.T.cell | Adaptive |
| CCL4 | Activated.CD4.T.cell | Adaptive |
| CCL5 | Activated.CD4.T.cell | Adaptive |
| CCNB1 | Activated.CD4.T.cell | Adaptive |
| CCR7 | Activated.CD4.T.cell | Adaptive |
| DUSP2 | Activated.CD4.T.cell | Adaptive |
| ESCO2 | Activated.CD4.T.cell | Adaptive |
| ETS1 | Activated.CD4.T.cell | Adaptive |
| EXO1 | Activated.CD4.T.cell | Adaptive |
| EXOC6 | Activated.CD4.T.cell | Adaptive |
| IARS | Activated.CD4.T.cell | Adaptive |
| ITK | Activated.CD4.T.cell | Adaptive |
| KIF11 | Activated.CD4.T.cell | Adaptive |
| KNTC1 | Activated.CD4.T.cell | Adaptive |
| NUF2 | Activated.CD4.T.cell | Adaptive |
| PRC1 | Activated.CD4.T.cell | Adaptive |
| PSAT1 | Activated.CD4.T.cell | Adaptive |
| RGS1 | Activated.CD4.T.cell | Adaptive |
| RTKN2 | Activated.CD4.T.cell | Adaptive |
| SAMSN1 | Activated.CD4.T.cell | Adaptive |
| SELL | Activated.CD4.T.cell | Adaptive |
| TRAT1 | Activated.CD4.T.cell | Adaptive |
| ADRM1 | Activated.CD8.T.cell | Adaptive |
| AHSA1 | Activated.CD8.T.cell | Adaptive |
| C1GALT1C1 | Activated.CD8.T.cell | Adaptive |
| CCT6B | Activated.CD8.T.cell | Adaptive |
| CD37 | Activated.CD8.T.cell | Adaptive |
| CD3D | Activated.CD8.T.cell | Adaptive |
| CD3E | Activated.CD8.T.cell | Adaptive |
| CD3G | Activated.CD8.T.cell | Adaptive |
| CD69 | Activated.CD8.T.cell | Adaptive |
| CD8A | Activated.CD8.T.cell | Adaptive |
| CETN3 | Activated.CD8.T.cell | Adaptive |
| CSE1L | Activated.CD8.T.cell | Adaptive |
| GEMIN6 | Activated.CD8.T.cell | Adaptive |
| GNLY | Activated.CD8.T.cell | Adaptive |
| GPT2 | Activated.CD8.T.cell | Adaptive |
| GZMA | Activated.CD8.T.cell | Adaptive |
| GZMH | Activated.CD8.T.cell | Adaptive |
| GZMK | Activated.CD8.T.cell | Adaptive |
| IL2RB | Activated.CD8.T.cell | Adaptive |
| LCK | Activated.CD8.T.cell | Adaptive |
| MPZL1 | Activated.CD8.T.cell | Adaptive |
| NKG7 | Activated.CD8.T.cell | Adaptive |
| PIK3IP1 | Activated.CD8.T.cell | Adaptive |
| PTRH2 | Activated.CD8.T.cell | Adaptive |
| TIMM13 | Activated.CD8.T.cell | Adaptive |
| ZAP70 | Activated.CD8.T.cell | Adaptive |
| ABCD1 | Activated.dendritic.cell | Adaptive |
| C1QC | Activated.dendritic.cell | Adaptive |
| CAPG | Activated.dendritic.cell | Adaptive |
| CCL3L3 | Activated.dendritic.cell | Adaptive |
| CD207 | Activated.dendritic.cell | Adaptive |
| CD302 | Activated.dendritic.cell | Adaptive |
| ATP5B | Activated.dendritic.cell | Adaptive |
| ATP5L | Activated.dendritic.cell | Adaptive |
| ATP6V1A | Activated.dendritic.cell | Adaptive |
| BCL2L1 | Activated.dendritic.cell | Adaptive |
| C1QB | Activated.dendritic.cell | Adaptive |
| SNURF | Activated.dendritic.cell | Adaptive |
| SPCS3 | Activated.dendritic.cell | Adaptive |
| CCNA1 | Activated.dendritic.cell | Adaptive |
| CEACAM8 | Activated.dendritic.cell | Adaptive |
| NOS2 | Activated.dendritic.cell | Adaptive |
| SRA1 | Activated.dendritic.cell | Adaptive |
| TNFRSF6B | Activated.dendritic.cell | Adaptive |
| TREM1 | Activated.dendritic.cell | Adaptive |
| TREML1 | Activated.dendritic.cell | Adaptive |
| RHOA | Activated.dendritic.cell | Adaptive |
| SLC25A37 | Activated.dendritic.cell | Adaptive |
| TNFSF14 | Activated.dendritic.cell | Adaptive |
| TREML4 | Activated.dendritic.cell | Adaptive |
| VNN2 | Activated.dendritic.cell | Adaptive |
| XPO6 | Activated.dendritic.cell | Adaptive |
| CLEC4C | Activated.dendritic.cell | Adaptive |
| TNFAIP2 | Activated.dendritic.cell | Adaptive |
| UBD | Activated.dendritic.cell | Adaptive |
| ACTR3 | Activated.dendritic.cell | Adaptive |
| RAB1A | Activated.dendritic.cell | Adaptive |
| SLA | Activated.dendritic.cell | Adaptive |
| HLA-DQA2 | Activated.dendritic.cell | Adaptive |
| SIGLEC5 | Activated.dendritic.cell | Adaptive |
| SLAMF9 | Activated.dendritic.cell | Adaptive |
| ACP5 | Gamma.delta.T.cell | Adaptive |
| AQP9 | Gamma.delta.T.cell | Adaptive |
| BTN3A2 | Gamma.delta.T.cell | Adaptive |
| C1orf54 | Gamma.delta.T.cell | Adaptive |
| CARD8 | Gamma.delta.T.cell | Adaptive |
| CCL18 | Gamma.delta.T.cell | Adaptive |
| CD209 | Gamma.delta.T.cell | Adaptive |
| CD33 | Gamma.delta.T.cell | Adaptive |
| CD36 | Gamma.delta.T.cell | Adaptive |
| CDK5 | Gamma.delta.T.cell | Adaptive |
| IL10RB | Gamma.delta.T.cell | Adaptive |
| KLRF1 | Gamma.delta.T.cell | Adaptive |
| LGALS1 | Gamma.delta.T.cell | Adaptive |
| MAPK7 | Gamma.delta.T.cell | Adaptive |
| KLHL7 | Gamma.delta.T.cell | Adaptive |
| KRT80 | Gamma.delta.T.cell | Adaptive |
| LAMC1 | Gamma.delta.T.cell | Adaptive |
| LCORL | Gamma.delta.T.cell | Adaptive |
| LMNB1 | Gamma.delta.T.cell | Adaptive |
| MEIS3P1 | Gamma.delta.T.cell | Adaptive |
| MPL | Gamma.delta.T.cell | Adaptive |
| FABP1 | Gamma.delta.T.cell | Adaptive |
| FABP5 | Gamma.delta.T.cell | Adaptive |
| FADD | Gamma.delta.T.cell | Adaptive |
| MFAP3L | Gamma.delta.T.cell | Adaptive |
| MINPP1 | Gamma.delta.T.cell | Adaptive |
| RPS24 | Gamma.delta.T.cell | Adaptive |
| RPS7 | Gamma.delta.T.cell | Adaptive |
| RPS9 | Gamma.delta.T.cell | Adaptive |
| DBNL | Gamma.delta.T.cell | Adaptive |
| CCL13 | Gamma.delta.T.cell | Adaptive |
| CD22 | Immature..B.cell | Adaptive |
| CYBB | Immature..B.cell | Adaptive |
| FAM129C | Immature..B.cell | Adaptive |
| FCRL1 | Immature..B.cell | Adaptive |
| FCRL3 | Immature..B.cell | Adaptive |
| FCRL5 | Immature..B.cell | Adaptive |
| FCRLA | Immature..B.cell | Adaptive |
| HDAC9 | Immature..B.cell | Adaptive |
| HLA-DQA1 | Immature..B.cell | Adaptive |
| HVCN1 | Immature..B.cell | Adaptive |
| KIAA0226 | Immature..B.cell | Adaptive |
| NCF1 | Immature..B.cell | Adaptive |
| NCF1B | Immature..B.cell | Adaptive |
| P2RY10 | Immature..B.cell | Adaptive |
| SP100 | Immature..B.cell | Adaptive |
| TXNIP | Immature..B.cell | Adaptive |
| STAP1 | Immature..B.cell | Adaptive |
| TAGAP | Immature..B.cell | Adaptive |
| ZCCHC2 | Immature..B.cell | Adaptive |
| CCL3L1 | Regulatory.T.cell | Adaptive |
| CD72 | Regulatory.T.cell | Adaptive |
| CLEC5A | Regulatory.T.cell | Adaptive |
| FOXP3 | Regulatory.T.cell | Adaptive |
| ITGA4 | Regulatory.T.cell | Adaptive |
| L1CAM | Regulatory.T.cell | Adaptive |
| LIPA | Regulatory.T.cell | Adaptive |
| LRP1 | Regulatory.T.cell | Adaptive |
| LRRC42 | Regulatory.T.cell | Adaptive |
| MARCO | Regulatory.T.cell | Adaptive |
| MMP12 | Regulatory.T.cell | Adaptive |
| MNDA | Regulatory.T.cell | Adaptive |
| MRC1 | Regulatory.T.cell | Adaptive |
| MS4A6A | Regulatory.T.cell | Adaptive |
| PELO | Regulatory.T.cell | Adaptive |
| PLEK | Regulatory.T.cell | Adaptive |
| PRSS23 | Regulatory.T.cell | Adaptive |
| PTGIR | Regulatory.T.cell | Adaptive |
| ST8SIA4 | Regulatory.T.cell | Adaptive |
| STAB1 | Regulatory.T.cell | Adaptive |
| B3GAT1 | T.follicular.helper.cell | Adaptive |
| CDK5R1 | T.follicular.helper.cell | Adaptive |
| PDCD1 | T.follicular.helper.cell | Adaptive |
| BCL6 | T.follicular.helper.cell | Adaptive |
| CD200 | T.follicular.helper.cell | Adaptive |
| CD83 | T.follicular.helper.cell | Adaptive |
| CD84 | T.follicular.helper.cell | Adaptive |
| FGF2 | T.follicular.helper.cell | Adaptive |
| GPR18 | T.follicular.helper.cell | Adaptive |
| CEBPA | T.follicular.helper.cell | Adaptive |
| CECR1 | T.follicular.helper.cell | Adaptive |
| CLEC10A | T.follicular.helper.cell | Adaptive |
| CLEC4A | T.follicular.helper.cell | Adaptive |
| CSF1R | T.follicular.helper.cell | Adaptive |
| CTSS | T.follicular.helper.cell | Adaptive |
| DMN | T.follicular.helper.cell | Adaptive |
| DPP4 | T.follicular.helper.cell | Adaptive |
| LRRC32 | T.follicular.helper.cell | Adaptive |
| MC5R | T.follicular.helper.cell | Adaptive |
| MICA | T.follicular.helper.cell | Adaptive |
| NCAM1 | T.follicular.helper.cell | Adaptive |
| NCR2 | T.follicular.helper.cell | Adaptive |
| NRP1 | T.follicular.helper.cell | Adaptive |
| PDCD1LG2 | T.follicular.helper.cell | Adaptive |
| PDCD6 | T.follicular.helper.cell | Adaptive |
| PRDX1 | T.follicular.helper.cell | Adaptive |
| RAE1 | T.follicular.helper.cell | Adaptive |
| RAET1E | T.follicular.helper.cell | Adaptive |
| SIGLEC7 | T.follicular.helper.cell | Adaptive |
| SIGLEC9 | T.follicular.helper.cell | Adaptive |
| TYRO3 | T.follicular.helper.cell | Adaptive |
| CHST12 | T.follicular.helper.cell | Adaptive |
| CLIC3 | T.follicular.helper.cell | Adaptive |
| IVNS1ABP | T.follicular.helper.cell | Adaptive |
| KIR2DL2 | T.follicular.helper.cell | Adaptive |
| LGMN | T.follicular.helper.cell | Adaptive |
| CD70 | Type.1.T.helper.cell | Adaptive |
| TBX21 | Type.1.T.helper.cell | Adaptive |
| ADAM8 | Type.1.T.helper.cell | Adaptive |
| AHCYL2 | Type.1.T.helper.cell | Adaptive |
| ALCAM | Type.1.T.helper.cell | Adaptive |
| B3GALNT1 | Type.1.T.helper.cell | Adaptive |
| BBS12 | Type.1.T.helper.cell | Adaptive |
| BST1 | Type.1.T.helper.cell | Adaptive |
| CD151 | Type.1.T.helper.cell | Adaptive |
| CD47 | Type.1.T.helper.cell | Adaptive |
| CD48 | Type.1.T.helper.cell | Adaptive |
| CD52 | Type.1.T.helper.cell | Adaptive |
| CD53 | Type.1.T.helper.cell | Adaptive |
| CD59 | Type.1.T.helper.cell | Adaptive |
| CD6 | Type.1.T.helper.cell | Adaptive |
| CD68 | Type.1.T.helper.cell | Adaptive |
| CD7 | Type.1.T.helper.cell | Adaptive |
| CD96 | Type.1.T.helper.cell | Adaptive |
| CFHR3 | Type.1.T.helper.cell | Adaptive |
| CHRM3 | Type.1.T.helper.cell | Adaptive |
| CLEC7A | Type.1.T.helper.cell | Adaptive |
| COL23A1 | Type.1.T.helper.cell | Adaptive |
| COL4A4 | Type.1.T.helper.cell | Adaptive |
| COL5A3 | Type.1.T.helper.cell | Adaptive |
| DAB1 | Type.1.T.helper.cell | Adaptive |
| DLEU7 | Type.1.T.helper.cell | Adaptive |
| DOC2B | Type.1.T.helper.cell | Adaptive |
| EMP1 | Type.1.T.helper.cell | Adaptive |
| F12 | Type.1.T.helper.cell | Adaptive |
| FURIN | Type.1.T.helper.cell | Adaptive |
| GAB3 | Type.1.T.helper.cell | Adaptive |
| GATM | Type.1.T.helper.cell | Adaptive |
| GFPT2 | Type.1.T.helper.cell | Adaptive |
| GPR25 | Type.1.T.helper.cell | Adaptive |
| GREM2 | Type.1.T.helper.cell | Adaptive |
| HAVCR1 | Type.1.T.helper.cell | Adaptive |
| HSD11B1 | Type.1.T.helper.cell | Adaptive |
| HUNK | Type.1.T.helper.cell | Adaptive |
| IGF2 | Type.1.T.helper.cell | Adaptive |
| RCSD1 | Type.1.T.helper.cell | Adaptive |
| RYR1 | Type.1.T.helper.cell | Adaptive |
| SAV1 | Type.1.T.helper.cell | Adaptive |
| SELE | Type.1.T.helper.cell | Adaptive |
| SELP | Type.1.T.helper.cell | Adaptive |
| SH3KBP1 | Type.1.T.helper.cell | Adaptive |
| SIT1 | Type.1.T.helper.cell | Adaptive |
| SLC35B3 | Type.1.T.helper.cell | Adaptive |
| SIGLEC10 | Type.1.T.helper.cell | Adaptive |
| SKAP1 | Type.1.T.helper.cell | Adaptive |
| THUMPD2 | Type.1.T.helper.cell | Adaptive |
| TIGIT | Type.1.T.helper.cell | Adaptive |
| ZEB2 | Type.1.T.helper.cell | Adaptive |
| ENC1 | Type.1.T.helper.cell | Adaptive |
| FAM134B | Type.1.T.helper.cell | Adaptive |
| FBXO30 | Type.1.T.helper.cell | Adaptive |
| FCGR2C | Type.1.T.helper.cell | Adaptive |
| STAC | Type.1.T.helper.cell | Adaptive |
| LTC4S | Type.1.T.helper.cell | Adaptive |
| MAN1B1 | Type.1.T.helper.cell | Adaptive |
| MDH1 | Type.1.T.helper.cell | Adaptive |
| MMD | Type.1.T.helper.cell | Adaptive |
| RGS16 | Type.1.T.helper.cell | Adaptive |
| IL12A | Type.1.T.helper.cell | Adaptive |
| P2RX5 | Type.1.T.helper.cell | Adaptive |
| CD97 | Type.1.T.helper.cell | Adaptive |
| ITGB4 | Type.1.T.helper.cell | Adaptive |
| ICAM3 | Type.1.T.helper.cell | Adaptive |
| METRNL | Type.1.T.helper.cell | Adaptive |
| TNFRSF1A | Type.1.T.helper.cell | Adaptive |
| IRF1 | Type.1.T.helper.cell | Adaptive |
| HTR2B | Type.1.T.helper.cell | Adaptive |
| CALD1 | Type.1.T.helper.cell | Adaptive |
| MOCOS | Type.1.T.helper.cell | Adaptive |
| TRAF3IP2 | Type.1.T.helper.cell | Adaptive |
| TLR8 | Type.1.T.helper.cell | Adaptive |
| TRAF1 | Type.1.T.helper.cell | Adaptive |
| DUSP14 | Type.1.T.helper.cell | Adaptive |
| IL17A | Type.17.T.helper.cell | Adaptive |
| IL17RA | Type.17.T.helper.cell | Adaptive |
| C2CD4A | Type.17.T.helper.cell | Adaptive |
| C2CD4B | Type.17.T.helper.cell | Adaptive |
| CA2 | Type.17.T.helper.cell | Adaptive |
| CCDC65 | Type.17.T.helper.cell | Adaptive |
| CEACAM3 | Type.17.T.helper.cell | Adaptive |
| IL17C | Type.17.T.helper.cell | Adaptive |
| IL17F | Type.17.T.helper.cell | Adaptive |
| IL17RC | Type.17.T.helper.cell | Adaptive |
| IL17RE | Type.17.T.helper.cell | Adaptive |
| IL23A | Type.17.T.helper.cell | Adaptive |
| ILDR1 | Type.17.T.helper.cell | Adaptive |
| LONRF3 | Type.17.T.helper.cell | Adaptive |
| SH2D6 | Type.17.T.helper.cell | Adaptive |
| TNIP2 | Type.17.T.helper.cell | Adaptive |
| ABCA1 | Type.17.T.helper.cell | Adaptive |
| ABCB1 | Type.17.T.helper.cell | Adaptive |
| ADAMTS12 | Type.17.T.helper.cell | Adaptive |
| ANK1 | Type.17.T.helper.cell | Adaptive |
| ANKRD22 | Type.17.T.helper.cell | Adaptive |
| B3GALT2 | Type.17.T.helper.cell | Adaptive |
| CAMTA1 | Type.17.T.helper.cell | Adaptive |
| CCR9 | Type.17.T.helper.cell | Adaptive |
| CD40 | Type.17.T.helper.cell | Adaptive |
| GPR44 | Type.17.T.helper.cell | Adaptive |
| IFT80 | Type.17.T.helper.cell | Adaptive |
| ASB2 | Type.2.T.helper.cell | Adaptive |
| CSRP2 | Type.2.T.helper.cell | Adaptive |
| DAPK1 | Type.2.T.helper.cell | Adaptive |
| DLC1 | Type.2.T.helper.cell | Adaptive |
| DNAJC12 | Type.2.T.helper.cell | Adaptive |
| DUSP6 | Type.2.T.helper.cell | Adaptive |
| GNAI1 | Type.2.T.helper.cell | Adaptive |
| LAMP3 | Type.2.T.helper.cell | Adaptive |
| NRP2 | Type.2.T.helper.cell | Adaptive |
| OSBPL1A | Type.2.T.helper.cell | Adaptive |
| PDE4B | Type.2.T.helper.cell | Adaptive |
| PHLDA1 | Type.2.T.helper.cell | Adaptive |
| PLA2G4A | Type.2.T.helper.cell | Adaptive |
| RAB27B | Type.2.T.helper.cell | Adaptive |
| RBMS3 | Type.2.T.helper.cell | Adaptive |
| RNF125 | Type.2.T.helper.cell | Adaptive |
| TMPRSS3 | Type.2.T.helper.cell | Adaptive |
| GATA3 | Type.2.T.helper.cell | Adaptive |
| BIRC5 | Type.2.T.helper.cell | Adaptive |
| CDC25C | Type.2.T.helper.cell | Adaptive |
| CDC7 | Type.2.T.helper.cell | Adaptive |
| CENPF | Type.2.T.helper.cell | Adaptive |
| CXCR6 | Type.2.T.helper.cell | Adaptive |
| DHFR | Type.2.T.helper.cell | Adaptive |
| EVI5 | Type.2.T.helper.cell | Adaptive |
| GSTA4 | Type.2.T.helper.cell | Adaptive |
| HELLS | Type.2.T.helper.cell | Adaptive |
| IL26 | Type.2.T.helper.cell | Adaptive |
| LAIR2 | Type.2.T.helper.cell | Adaptive |
| ABAT | CD56bright.natural.killer.cell | Innate |
| C11orf75 | CD56bright.natural.killer.cell | Innate |
| C5orf15 | CD56bright.natural.killer.cell | Innate |
| CDHR1 | CD56bright.natural.killer.cell | Innate |
| DCAF12 | CD56bright.natural.killer.cell | Innate |
| DYNLL1 | CD56bright.natural.killer.cell | Innate |
| GPR137B | CD56bright.natural.killer.cell | Innate |
| HCP5 | CD56bright.natural.killer.cell | Innate |
| HDGFRP2 | CD56bright.natural.killer.cell | Innate |
| KRT86 | CD56bright.natural.killer.cell | Innate |
| MLST8 | CD56bright.natural.killer.cell | Innate |
| ELMOD3 | CD56bright.natural.killer.cell | Innate |
| ENTPD5 | CD56bright.natural.killer.cell | Innate |
| FAM119A | CD56bright.natural.killer.cell | Innate |
| FAM179A | CD56bright.natural.killer.cell | Innate |
| CLIC2 | CD56bright.natural.killer.cell | Innate |
| COX7A2L | CD56bright.natural.killer.cell | Innate |
| CREB3L4 | CD56bright.natural.killer.cell | Innate |
| CSF1 | CD56bright.natural.killer.cell | Innate |
| CSNK2A2 | CD56bright.natural.killer.cell | Innate |
| CSTA | CD56bright.natural.killer.cell | Innate |
| CSTB | CD56bright.natural.killer.cell | Innate |
| CTPS | CD56bright.natural.killer.cell | Innate |
| CTSD | CD56bright.natural.killer.cell | Innate |
| FST | CD56bright.natural.killer.cell | Innate |
| GATA2 | CD56bright.natural.killer.cell | Innate |
| GMPR | CD56bright.natural.killer.cell | Innate |
| HDC | CD56bright.natural.killer.cell | Innate |
| HEY1 | CD56bright.natural.killer.cell | Innate |
| HOXA1 | CD56bright.natural.killer.cell | Innate |
| HS2ST1 | CD56bright.natural.killer.cell | Innate |
| HS3ST1 | CD56bright.natural.killer.cell | Innate |
| BCL11B | CD56bright.natural.killer.cell | Innate |
| CDH3 | CD56bright.natural.killer.cell | Innate |
| MYL6B | CD56bright.natural.killer.cell | Innate |
| NAA16 | CD56bright.natural.killer.cell | Innate |
| ClQA | CD56bright.natural.killer.cell | Innate |
| ClQB | CD56bright.natural.killer.cell | Innate |
| CYP27B1 | CD56bright.natural.killer.cell | Innate |
| EIF3M | CD56bright.natural.killer.cell | Innate |
| CYP27A1 | CD56dim.natural.killer.cell | Innate |
| DDX55 | CD56dim.natural.killer.cell | Innate |
| DYRK2 | CD56dim.natural.killer.cell | Innate |
| RPL37A | CD56dim.natural.killer.cell | Innate |
| NOTCH3 | CD56dim.natural.killer.cell | Innate |
| AKR7A3 | CD56dim.natural.killer.cell | Innate |
| GPRC5C | CD56dim.natural.killer.cell | Innate |
| GRIN1 | CD56dim.natural.killer.cell | Innate |
| HLA-E | CD56dim.natural.killer.cell | Innate |
| PORCN | CD56dim.natural.killer.cell | Innate |
| PSMC4 | CD56dim.natural.killer.cell | Innate |
| UPP1 | CD56dim.natural.killer.cell | Innate |
| IL21R | CD56dim.natural.killer.cell | Innate |
| KIR2DS1 | CD56dim.natural.killer.cell | Innate |
| KIR2DS2 | CD56dim.natural.killer.cell | Innate |
| KIR2DS5 | CD56dim.natural.killer.cell | Innate |
| GIPR | Eosinophilna | Innate |
| KRT18P50 | Eosinophilna | Innate |
| LRMP | Eosinophilna | Innate |
| FOSB | Eosinophilna | Innate |
| RRP12 | Eosinophilna | Innate |
| GPR183 | Eosinophilna | Innate |
| NR4A3 | Eosinophilna | Innate |
| ST3GAL6 | Eosinophilna | Innate |
| DEPDC5 | Eosinophilna | Innate |
| PDE6C | Eosinophilna | Innate |
| PKD2L2 | Eosinophilna | Innate |
| GPR65 | Eosinophilna | Innate |
| IL5RA | Eosinophilna | Innate |
| P2RY14 | Eosinophilna | Innate |
| DACH1 | Eosinophilna | Innate |
| DAPK2 | Eosinophilna | Innate |
| EMR3 | Eosinophilna | Innate |
| ACADM | Immature.dendritic.cell | Innate |
| AHCYL1 | Immature.dendritic.cell | Innate |
| ALDH1A2 | Immature.dendritic.cell | Innate |
| ALDH3A2 | Immature.dendritic.cell | Innate |
| ALDH9A1 | Immature.dendritic.cell | Innate |
| ALOX15 | Immature.dendritic.cell | Innate |
| AMT | Immature.dendritic.cell | Innate |
| ARL1 | Immature.dendritic.cell | Innate |
| ATIC | Immature.dendritic.cell | Innate |
| ATP5A1 | Immature.dendritic.cell | Innate |
| CAPZA1 | Immature.dendritic.cell | Innate |
| LILRA5 | Immature.dendritic.cell | Innate |
| RDX | Immature.dendritic.cell | Innate |
| RRAGD | Immature.dendritic.cell | Innate |
| TACSTD2 | Immature.dendritic.cell | Innate |
| INPP5F | Immature.dendritic.cell | Innate |
| RAB38 | Immature.dendritic.cell | Innate |
| PLAU | Immature.dendritic.cell | Innate |
| CSF3R | Immature.dendritic.cell | Innate |
| SLC18A2 | Immature.dendritic.cell | Innate |
| AMPD2 | Immature.dendritic.cell | Innate |
| CLTB | Immature.dendritic.cell | Innate |
| C1orf162 | Immature.dendritic.cell | Innate |
| CCR2 | MDSC | Innate |
| CD14 | MDSC | Innate |
| CD2 | MDSC | Innate |
| CD86 | MDSC | Innate |
| CXCR4 | MDSC | Innate |
| FCGR2A | MDSC | Innate |
| FCGR2B | MDSC | Innate |
| FCGR3A | MDSC | Innate |
| FERMT3 | MDSC | Innate |
| GPSM3 | MDSC | Innate |
| IL18BP | MDSC | Innate |
| IL4R | MDSC | Innate |
| ITGAL | MDSC | Innate |
| ITGAM | MDSC | Innate |
| PARVG | MDSC | Innate |
| PSAP | MDSC | Innate |
| PTGER2 | MDSC | Innate |
| PTGES2 | MDSC | Innate |
| S100A8 | MDSC | Innate |
| S100A9 | MDSC | Innate |
| AIF1 | Macrophagena | Innate |
| CCL1 | Macrophagena | Innate |
| CCL14 | Macrophagena | Innate |
| CCL23 | Macrophagena | Innate |
| CCL26 | Macrophagena | Innate |
| CD300LB | Macrophagena | Innate |
| CNR1 | Macrophagena | Innate |
| CNR2 | Macrophagena | Innate |
| EIF1 | Macrophagena | Innate |
| EIF4A1 | Macrophagena | Innate |
| FPR1 | Macrophagena | Innate |
| FPR2 | Macrophagena | Innate |
| FRAT2 | Macrophagena | Innate |
| GPR27 | Macrophagena | Innate |
| GPR77 | Macrophagena | Innate |
| RNASE2 | Macrophagena | Innate |
| MS4A2 | Macrophagena | Innate |
| BASP1 | Macrophagena | Innate |
| IGSF6 | Macrophagena | Innate |
| HK3 | Macrophagena | Innate |
| VNN1 | Macrophagena | Innate |
| FES | Macrophagena | Innate |
| NPL | Macrophagena | Innate |
| FZD2 | Macrophagena | Innate |
| FAM198B | Macrophagena | Innate |
| HNMT | Macrophagena | Innate |
| SLC15A3 | Macrophagena | Innate |
| CD4 | Macrophagena | Innate |
| TXNDC3 | Macrophagena | Innate |
| FRMD4A | Macrophagena | Innate |
| CRYBB1 | Macrophagena | Innate |
| HRH1 | Macrophagena | Innate |
| WNT5B | Macrophagena | Innate |
| ADAMTS3 | Mast.cell | Innate |
| CPA3 | Mast.cell | Innate |
| CMA1 | Mast.cell | Innate |
| CTSG | Mast.cell | Innate |
| ARHGAP15 | Mast.cell | Innate |
| CPM | Mast.cell | Innate |
| FCN1 | Mast.cell | Innate |
| FTL | Mast.cell | Innate |
| HSPA6 | Mast.cell | Innate |
| ITGA9 | Mast.cell | Innate |
| RNASE3 | Mast.cell | Innate |
| S100A4 | Mast.cell | Innate |
| SIGLEC8 | Mast.cell | Innate |
| SLC6A4 | Mast.cell | Innate |
| PTGS2 | Mast.cell | Innate |
| EGR3 | Mast.cell | Innate |
| PILRA | Mast.cell | Innate |
| ASGR2 | Monocyte | Innate |
| CFP | Monocyte | Innate |
| ASGR1 | Monocyte | Innate |
| CD1D | Monocyte | Innate |
| UPK3A | Monocyte | Innate |
| ACTG1 | Monocyte | Innate |
| ANXA5 | Monocyte | Innate |
| ATP6V1B2 | Monocyte | Innate |
| CFL1 | Monocyte | Innate |
| DAZAP2 | Monocyte | Innate |
| CTBS | Monocyte | Innate |
| EMR4P | Monocyte | Innate |
| HIVEP2 | Monocyte | Innate |
| MARCKSL1 | Monocyte | Innate |
| MBP | Monocyte | Innate |
| MMP15 | Monocyte | Innate |
| PNPLA6 | Monocyte | Innate |
| TMBIM6 | Monocyte | Innate |
| PQBP1 | Monocyte | Innate |
| TEX264 | Monocyte | Innate |
| IKZF1 | Monocyte | Innate |
| BTN2A2 | Natural.killer.T.cell | Innate |
| CD101 | Natural.killer.T.cell | Innate |
| CD109 | Natural.killer.T.cell | Innate |
| CNPY3 | Natural.killer.T.cell | Innate |
| CNPY4 | Natural.killer.T.cell | Innate |
| CREB1 | Natural.killer.T.cell | Innate |
| CRTC2 | Natural.killer.T.cell | Innate |
| CRTC3 | Natural.killer.T.cell | Innate |
| CSF2 | Natural.killer.T.cell | Innate |
| KLRC1 | Natural.killer.T.cell | Innate |
| FUT4 | Natural.killer.T.cell | Innate |
| ICAM2 | Natural.killer.T.cell | Innate |
| IL32 | Natural.killer.T.cell | Innate |
| LAMP2 | Natural.killer.T.cell | Innate |
| LILRB5 | Natural.killer.T.cell | Innate |
| KLRG1 | Natural.killer.T.cell | Innate |
| HSPA4 | Natural.killer.T.cell | Innate |
| HSPB6 | Natural.killer.T.cell | Innate |
| ISM2 | Natural.killer.T.cell | Innate |
| ITIH2 | Natural.killer.T.cell | Innate |
| KDM4C | Natural.killer.T.cell | Innate |
| KIR2DS4 | Natural.killer.T.cell | Innate |
| KIRREL3 | Natural.killer.T.cell | Innate |
| SDCBP | Natural.killer.T.cell | Innate |
| NFATC2IP | Natural.killer.T.cell | Innate |
| MICB | Natural.killer.T.cell | Innate |
| KIR2DL1 | Natural.killer.T.cell | Innate |
| KIR2DL3 | Natural.killer.T.cell | Innate |
| KIR3DL1 | Natural.killer.T.cell | Innate |
| KIR3DL2 | Natural.killer.T.cell | Innate |
| NCR1 | Natural.killer.T.cell | Innate |
| FOSL1 | Natural.killer.T.cell | Innate |
| TSLP | Natural.killer.T.cell | Innate |
| SLC7A7 | Natural.killer.T.cell | Innate |
| SPP1 | Natural.killer.T.cell | Innate |
| TREM2 | Natural.killer.T.cell | Innate |
| UBASH3A | Natural.killer.T.cell | Innate |
| YBX2 | Natural.killer.T.cell | Innate |
| CCDC88A | Natural.killer.T.cell | Innate |
| CLEC1A | Natural.killer.T.cell | Innate |
| THBD | Natural.killer.T.cell | Innate |
| PDPN | Natural.killer.T.cell | Innate |
| VCAM1 | Natural.killer.T.cell | Innate |
| EMR1 | Natural.killer.T.cell | Innate |
| AKT3 | Natural.killer.cell | Innate |
| AXL | Natural.killer.cell | Innate |
| BST2 | Natural.killer.cell | Innate |
| CDH2 | Natural.killer.cell | Innate |
| CRTAM | Natural.killer.cell | Innate |
| CSF2RA | Natural.killer.cell | Innate |
| CTSZ | Natural.killer.cell | Innate |
| CXCL1 | Natural.killer.cell | Innate |
| CYTH1 | Natural.killer.cell | Innate |
| DAXX | Natural.killer.cell | Innate |
| DGKH | Natural.killer.cell | Innate |
| DLL4 | Natural.killer.cell | Innate |
| DPYD | Natural.killer.cell | Innate |
| ERBB3 | Natural.killer.cell | Innate |
| F11R | Natural.killer.cell | Innate |
| FAM27A | Natural.killer.cell | Innate |
| FAM49A | Natural.killer.cell | Innate |
| FASLG | Natural.killer.cell | Innate |
| FCGR1A | Natural.killer.cell | Innate |
| FN1 | Natural.killer.cell | Innate |
| FSTL1 | Natural.killer.cell | Innate |
| FUCA1 | Natural.killer.cell | Innate |
| GBP3 | Natural.killer.cell | Innate |
| GLS2 | Natural.killer.cell | Innate |
| GRB2 | Natural.killer.cell | Innate |
| LST1 | Natural.killer.cell | Innate |
| BCL2 | Natural.killer.cell | Innate |
| CDC5L | Natural.killer.cell | Innate |
| FGF18 | Natural.killer.cell | Innate |
| FUT5 | Natural.killer.cell | Innate |
| FZR1 | Natural.killer.cell | Innate |
| GAGE2 | Natural.killer.cell | Innate |
| IGFBP5 | Natural.killer.cell | Innate |
| KANK2 | Natural.killer.cell | Innate |
| LDB3 | Natural.killer.cell | Innate |
| CREB5 | Neutrophil | Innate |
| CDA | Neutrophil | Innate |
| CHST15 | Neutrophil | Innate |
| S100A12 | Neutrophil | Innate |
| APOBEC3A | Neutrophil | Innate |
| CASP5 | Neutrophil | Innate |
| MMP25 | Neutrophil | Innate |
| HAL | Neutrophil | Innate |
| C1orf183 | Neutrophil | Innate |
| FFAR2 | Neutrophil | Innate |
| MAK | Neutrophil | Innate |
| CXCR1 | Neutrophil | Innate |
| STEAP4 | Neutrophil | Innate |
| MGAM | Neutrophil | Innate |
| BTNL8 | Neutrophil | Innate |
| CXCR2 | Neutrophil | Innate |
| TNFRSF10C | Neutrophil | Innate |
| VNN3 | Neutrophil | Innate |
| CBX6 | Plasmacytoid.dendritic.cell | Innate |
| DAB2 | Plasmacytoid.dendritic.cell | Innate |
| DDX17 | Plasmacytoid.dendritic.cell | Innate |
| HIGD1A | Plasmacytoid.dendritic.cell | Innate |
| IDH3A | Plasmacytoid.dendritic.cell | Innate |
| IL3RA | Plasmacytoid.dendritic.cell | Innate |
| MAGED1 | Plasmacytoid.dendritic.cell | Innate |
| NUCB2 | Plasmacytoid.dendritic.cell | Innate |
| OFD1 | Plasmacytoid.dendritic.cell | Innate |
| OGT | Plasmacytoid.dendritic.cell | Innate |
| PDIA4 | Plasmacytoid.dendritic.cell | Innate |
| SERTAD2 | Plasmacytoid.dendritic.cell | Innate |
| SIRPA | Plasmacytoid.dendritic.cell | Innate |
| TMED2 | Plasmacytoid.dendritic.cell | Innate |
| ENG | Plasmacytoid.dendritic.cell | Innate |
| FCAR | Plasmacytoid.dendritic.cell | Innate |
| IGF1 | Plasmacytoid.dendritic.cell | Innate |
| ITGA2B | Plasmacytoid.dendritic.cell | Innate |
| GABARAP | Plasmacytoid.dendritic.cell | Innate |
| GPX1 | Plasmacytoid.dendritic.cell | Innate |
| KRT23 | Plasmacytoid.dendritic.cell | Innate |
| PROK2 | Plasmacytoid.dendritic.cell | Innate |
| RALB | Plasmacytoid.dendritic.cell | Innate |
| RETNLB | Plasmacytoid.dendritic.cell | Innate |
| RNF141 | Plasmacytoid.dendritic.cell | Innate |
| SEC14L1 | Plasmacytoid.dendritic.cell | Innate |
| SEPX1 | Plasmacytoid.dendritic.cell | Innate |
| EMP3 | Plasmacytoid.dendritic.cell | Innate |
| CD300LF | Plasmacytoid.dendritic.cell | Innate |
| ABTB1 | Plasmacytoid.dendritic.cell | Innate |
| KLHL21 | Plasmacytoid.dendritic.cell | Innate |
| PHRF1 | Plasmacytoid.dendritic.cell | Innate |
